# Spectroelectrochemistry with Ultrathin Ion-Selective Membranes: Three Distinct Ranges for Analytical Sensing

**DOI:** 10.1021/acs.analchem.2c01584

**Published:** 2022-06-10

**Authors:** Yujie Liu, Gastón A. Crespo, María Cuartero

**Affiliations:** Department of Chemistry, School of Engineering Science in Chemistry, Biotechnology and Health, KTH Royal Institute of Technology, Teknikringen 30, SE-100 44 Stockholm, Sweden

## Abstract

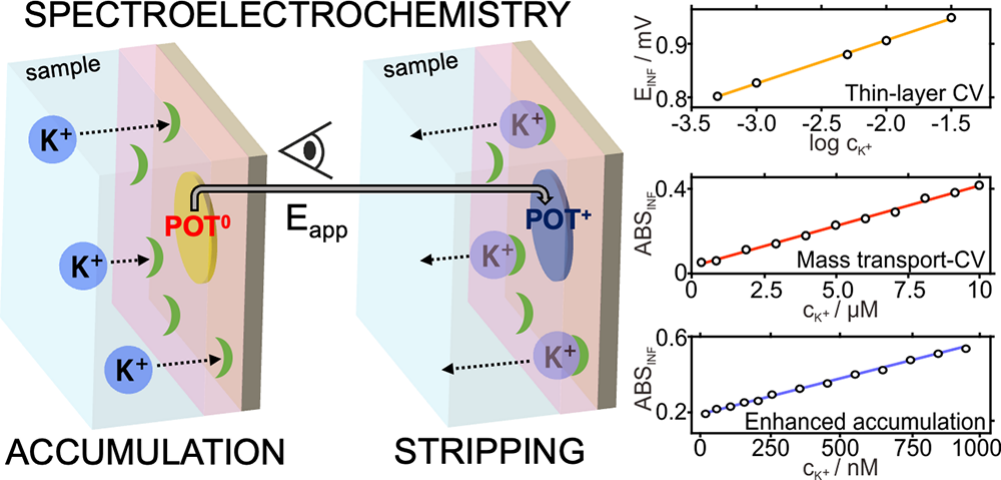

We present spectroelectrochemical sensing of the potassium ion (K^+^) at three very distinct analytical ranges—nanomolar, micromolar, and millimolar—when using the same ion-selective electrode (ISE) but interrogated under various regimes. The ISE is conceived in the all-solid-state format: an ITO glass modified with the conducting polymer poly(3-octylethiophene) (POT) and an ultrathin potassium-selective membrane. The experimental setup is designed to apply a potential in a three-electrode electrochemical cell with the ISE as the working electrode, while dynamic spectral changes in the POT film are simultaneously registered. The POT film is gradually oxidized to POT^+^, and this process is ultimately linked to K^+^ transfer at the membrane-sample interface, attending to electroneutrality requirements. The spectroelectrochemistry experiment provides two signals: a voltammetric peak and a transient absorbance response, with the latter of special interest because of its correspondence with the generated charge in the POT and thus with the ionic charge expelled from the membrane. By modifying how the ion analyte (K^+^ but also others) is initially accumulated into the membrane, we found three ranges of response for the absorbance: 10–950 nM for an accumulation-stripping protocol, 0.5–10 μM in diffusion-controlled cyclic voltammetry, and 0.5–32 mM with thin-layer cyclic voltammetry. This wide response range is a unique feature, one that is rare to find for a sensor and indeed for any analytical technique. Accordingly, the developed sensor is highly appealing for many analytical applications, especially considering the versatility of samples and ion analytes that may be spotted.

In recent years, increasing progress has been made in the research field of ion-selective electrodes (ISEs) based on polymeric ion-selective membranes (ISMs) that are interrogated using dynamic electrochemical techniques.^1^ The measurements go beyond the traditional zero-current interrogation in potentiometry to pursue challenging analyses based on calibration-free sensors,^2^ titration of confined samples,^3^ the improvement of the limit of detection,^4^ the simultaneous determination of multiple analytes,^5^ high-concentration discrimination,^6^ and the extension of the linear range of response,^7^ among others. Principally, the use of all-solid-state electrodes based on a conducting polymer as the ion-to-electron transducer material with the ISM on top (i.e., a double-layer design) seems to stand out over other design options. In essence, the ion-to-electron transducer is utilized to generate a charge disbalance in the electrode via an electrical perturbation that, in the end, results in an ion transfer (IT) at the membrane-sample interface.

The transducer must be redox-active while compatible with a reversible (un)doping process involving ionic species present in the membrane and/or coming from the solution. Such a charge-based mechanism has been mainly demonstrated with the conducting polymers poly(3,4-ethylene dioxythiophene)^8,9^ and poly(3-octylthiophene) (POT),^10,11^ but other materials have also been explored, including ferrocene self-assembling monolayers^12,13^ as well as metallic compounds and helicenes directly solved in the ISM.^14−17^ In particular for POT, and with electroneutrality as the driving force of the global process, it was evidenced that an anodic potential sweep provokes the oxidation of its basal state POT^0^ to POT^+^, which is stabilized by the lipophilic anion in the cation exchanger in the membrane (tetrakis[3,5-bis(trifluoromethyl)phenyl]borate, TFPB^–^) and generates the release of the cationic counterpart (sodium ion, Na^+^) from the membrane to the solution.^10^ This latter IT can be conveniently controlled for different analytical purposes.

The ISM may contain up to three cation ionophores that make the transfer selective for certain ions. The simultaneous incorporation of lithium, sodium, and potassium ionophores in the ISM makes it possible to obtain individual peaks for each cation and was used in the analysis of undiluted authentic human blood.^18^ Silver-selective membranes formulated with a reduced exchange capacity and interrogated under an accumulation/stripping protocol were demonstrated to be responsive at nanomolar concentrations in water samples.^4,19^ Notably, the thickness of the membrane used in these successful cases was claimed to be crucial to preventing any mass transport limitation on charge carrier rearrangement within the membrane domain: ca. 250 nm.^10^

Recently, spectroelectrochemical experiments were proposed to monitor the POT oxidation process in connection with the IT across different membranes.^20^ The absorbance changes registered when converting POT^0^ to POT^+^ are ascribable to the electron transfer (ET) in the film, which is visualized as a sigmoid curve in different potential windows. In membranes presenting several ITs, the entire ET profile manifests in different sigmoid-shaped parts connected to every IT. The key discovery was that the dynamic absorbance of the POT film unequivocally represents the connected IT(s). The mathematical Sigmoidal–Boltzmann distribution was found to fairly model the system, with key parameters, such as the standard potential for the IT and the average number of electrons involved in the ET, being implied in the equation. All the ITs can be combined in one mathematical expression in the form of the following equation (eq 1):^20,21^
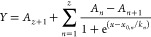
1where and expresses the dynamic oxidation of POT in relation to the cation exchanger content (*c*_TFPB^–^_^0^), *x* is equal to the applied potential (*E*_app_), *z* is the number of exchangeable ions present in the membrane, *A* values are distribution parameters that weigh each mathematical term (and therefore the contribution of each IT to the overall sigmoidal curve), *x*_0,*n*_ values are the potentials for each IT, and *k_n_* values are related to the ET in the POT lattice in connection to each IT ( , with *F* being the Faraday constant, *R* the gas constant, *T* the absolute temperature, and *n*_POT_ the average number of electrons transferred at the electrode surface to go from POT^0^ to POT^+^).

In this work, we demonstrate spectroelectrochemical sensing of the potassium ion (K^+^), as a model target, at three very distinct analytical ranges and using the same POT-membrane ISE. The tuning of the accumulation input of K^+^ into the membrane enables such a unique analytical feature, while changes in absorbance are dynamically monitored. Accumulation-stripping voltammetry allows for K^+^ detection at the nanomolar levels, whereas diffusion-controlled cyclic voltammetry and thin-layer cyclic voltammetry target micromolar and millimolar K^+^, respectively. Semi-empirical calculations predict and confirm the discovered behavior. The wide concentration range of response is highly appealing to the analytical chemistry domain, especially regarding the versatility of samples that could be spotted. Moreover, the developed methodology may be easily applied to other ions by exchanging the ionophore in the ultrathin ISM.

## Experimental Section

**Figure 1:**
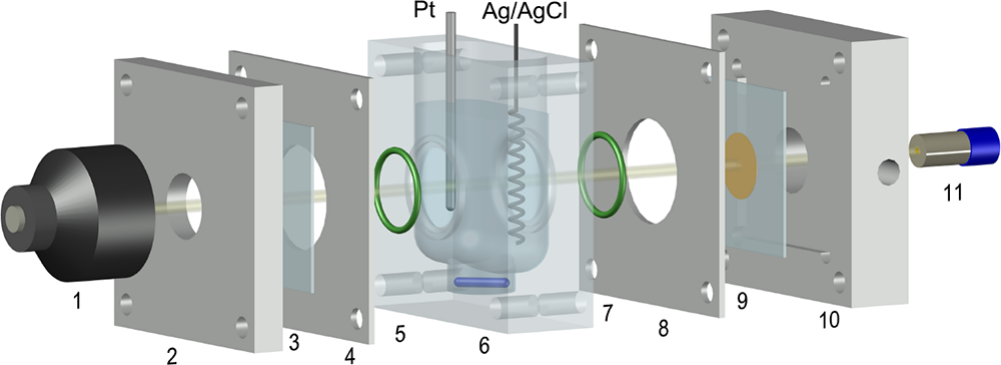
Spectroelectrochemical setup composed of (1) the light detector, (2 and 10) metallic holders, (3) the ITO, (4 and 8) metallic spacers, (5 and 7) o-rings, (6) the acrylic sample compartment, (9) the ITO-POT-membrane working electrode, and (11) the light source, a counter electrode (Pt rod), a reference electrode (Ag/AgCl wire), and a stirring bar.

The ITO-POT-membrane electrode was prepared, as described in the Supporting Information. Briefly, the POT film was obtained via electropolymerization. The membrane was selective for K^+^, with an ultrathin configuration (ca. 250 nm) comprising Na^+^TFPB^–^ as the cation exchanger, a K^+^ ionophore, a polymer, and a plasticizer. The design of the spectroelectrochemical cell is shown in Figure 1. The ITO-POT-membrane acts as the working electrode and is placed between a metallic part and spacer. A glass window makes up the opposite side of the cell using a similar configuration. An acrylic reservoir with a stirring compartment in the bottom enables the loading and stirring of ca. 7 mL sample volume without affecting the optical measurement, which occurs in the horizontal direction. From the backside of the working electrode, the light source is introduced, generating a beam that goes through the sample solution and finally reaches the detector placed behind the glass window (the second ITO). During spectroelectrochemical measurements, both the counter electrode and the reference electrode are placed in the sample solution beside the light beam. Figure S1 in the Supporting Information shows a real picture of the experimental setup.

## Results and Discussion

We herein present our findings on the sensing capability of ISEs based on ultrathin ion-selective membranes as determined through spectroelectrochemical measurements. The approach comprises an ITO-POT-membrane electrode working on a charge transfer (CT) principle with POT as the core element due to a change in its absorbance (at 450 nm) while being oxidized. Using the same ISE, we investigated how to displace the linear range of response of the optical signal attending to different working mechanisms, covering levels from millimolar to nanomolar. Fundamentally, any operational regime of the ISE involves electroneutrality as the driving force of the global process: An anodic potential sweep provokes the gradual oxidation of the basal state of the POT^0^ film to POT^+^, which is stabilized with the TFPB^–^ in the membrane and eventually generates the release of any cation from the membrane to the solution. The key aspect is which cation(s) is(are) present in the membrane and the path that enables its(their) presence there (i.e., the accumulation step). Thus, with Na^+^ coming from Na^+^TFPB^–^ (cation-exchanger), the only cation initially present in the membrane (as per its preparation), from the very first moment at which the membrane is in contact with the solution containing the cation analyte (in our case, K^+^), there is a gradual partial/total replacement of Na^+^ for K^+^ that mainly depends on (i) the concentration of K^+^ in the bulk solution and (ii) the conditions for the mass transport from the solution to the membrane. Tailoring these two factors, we reach different response ranges. The global accumulation/stripping mechanism as just described is presented in Figure S2.

The response range targeting concentrations from millimolar to nanomolar levels is a unique feature, rare to be found for a sensor and even for any analytical technique. For example, considering potentiometric ISEs that are indeed claimed to present a wide range of response as a regular feature, while a common linear range of response is from 10 μM to 10 mM ion concentration (or formally activity), dozen nanomolar levels can be fundamentally reached in inner-filling solution ISEs formulated under specific conditions to control ion fluxes. Such a concept was successfully demonstrated in the early 2000s but never reaching sufficient maturity to be applied in authentic analytical cases, in contrast to the analytical concept here developed.^22^

### Operational Mode 1. Cyclic Voltammetry at Millimolar K^+^ Levels: Thin-Layer Regime

**Figure 2:**
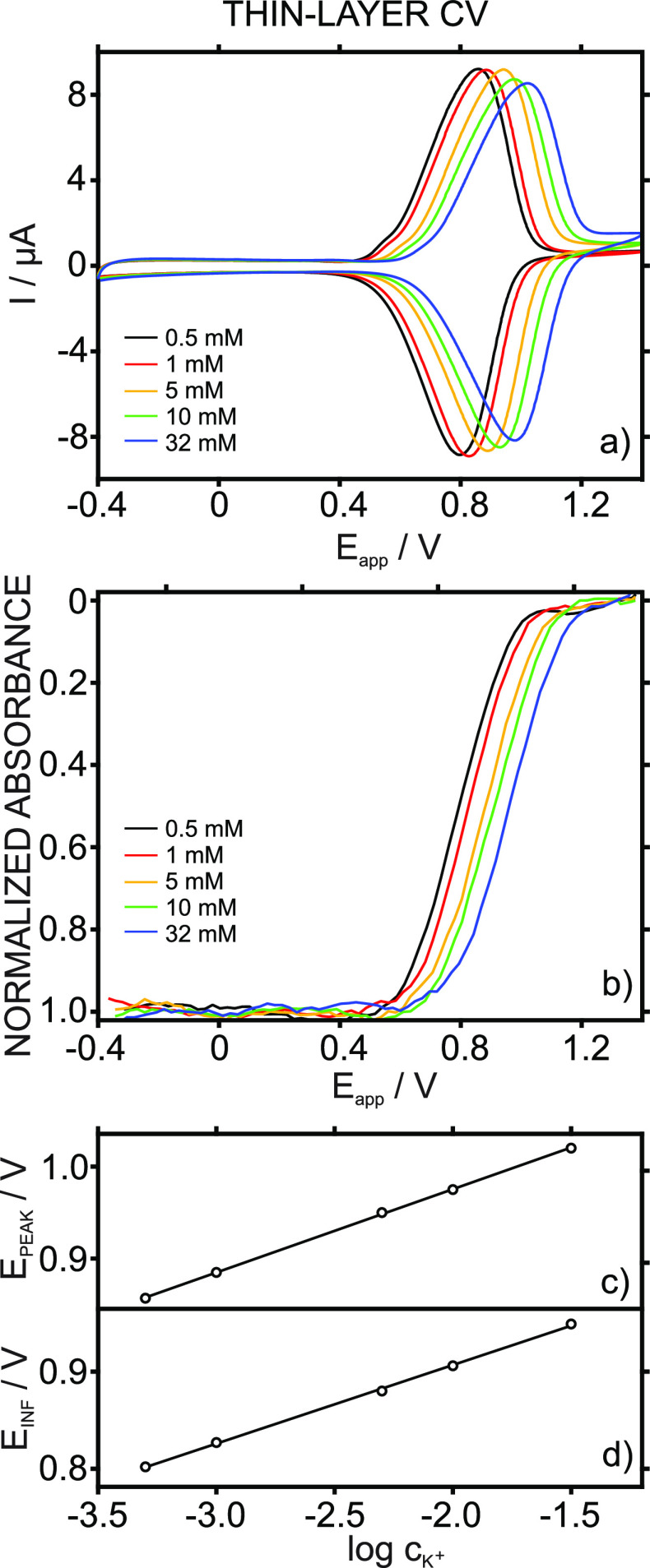
Experiments at the millimolar K^+^ concentration levels. (a) Cyclic voltammograms at increasing KCl concentrations with a 10 mM NaCl background. (b) Dynamic absorbance curves related to the anodic part of the voltammograms. Corresponding calibration curves using the (c) peak current and (d) potential at the inflection point of the dynamic absorbance curve. Scan rate: 50 mV s^–1^. Wavelength: 450 nm.

Spectroelectrochemical experiments were performed with the ITO-POT-membrane at increasing KCl concentrations, in the range from 0.5 to 32 mM, with a 10 mM NaCl background. The electrode was interrogated under cyclic voltammetry (CV) in the potential window from −0.4 to 1.3 V (Figure 2a), and in parallel, the absorbance of the POT film was dynamically acquired in the range of 400–500 nm, selecting 450 nm as the studied wavelength (Figure 2b). No time for the accumulation step was provided before starting the experiment. At a millimolar concentration of K^+^, its mass transport in the solution is not the rate-limiting step for the global CT-based mechanism. Moreover, the equilibration time for the membrane is roughly estimated to be in the order of tens of milliseconds, for a thickness of 200 nm, and a common diffusion coefficient of 10^–8^ cm^2^ s^–1^.^23,24^ CVs were acquired right after the K^+^ concentration was increased in the sample (ca. 30 s of stirring after each K^+^ addition to ensure solution homogeneity).

The CVs at increasing K^+^ concentrations displayed one voltammetric peak, which was ascribed to K^+^ expelling from the membrane, and that shifted to more positive potentials (Figure 2a). Such a displacement exhibited linearity with the logarithmic K^+^ concentration, as can be observed in Figure 2c [*E*_PEAK_(mV) = 87.63 log *c*_K^+^_(mM) + 1146.5, *R*^2^ = 0.9993]. The CV peaks were found to be fairly reversible and, therefore, so is the overall working principle: with an average separation between the position of the anodic and cathodic waves of |*E*_anodic_ – *E*_cathodic_| = 52 ± 8 mV and a peak charge that is very similar for the anodic and cathodic waves, 48.5 ± 2.6 and 51.4 ± 0.9 μC, respectively. It is notable that, as K^+^ is increased in the solution, the voltammetric peak is slightly wider (*w*_1/2_ increasing from 280 to 300 mV for 0.5 and 32 mM K^+^ concentrations) and develops a certain ohmic drop in the zone of the peak growth that is likely related to an increase in the resistance of the IT. In essence, the K^+^ expelling process from the membrane to the solution presents higher resistance as the K^+^ concentration in the solution increases.

The dynamic absorbance curves during the anodic scan at increasing K^+^ concentrations (Figure 2b) displayed sigmoidal shapes coinciding with the potential window of the corresponding voltammetric peak (Table S1). The absorbance of the sigmoid goes from 1 to 0 after the values are normalized considering that POT^0^ (basal state before applying the potential) presents the maximum absorbance at 450 nm and that this diminishes while POT^0^ is oxidized to POT^+^. Then, the minimum absorbance is reached when the POT^+^ charge equals the TFPB^–^ in the membrane. An example of untreated absorbance data is provided in Figure S3. Notably, in the ITO-POT-membrane system, the amount of TFPB^–^ limits the total charge that can be generated (and doped) in the POT film and, therefore, the maximum color change that can be reached.^21^ For simplicity, only the anodic part is used throughout the paper, with the cathodic part being a mirror image (see Figure S4a). The potential at which the inflection point appears in each sigmoid (*E*_INF_), which corresponds to a normalized absorbance equal to 0.5, was found to agree with the peak potentials and also linearly shifted with the logarithmic K^+^ concentration (Figure 2d): *E*_INF_(mV) = 80.63 log *c*_K^+^_(mM) + 826.1, *R*^2^ = 0.9993. The reversibility of the observed dynamic absorbance was fairly good, and so, the reproducibility for the calculated potentials at the inflection points over voltammetric cycles (824.4 ± 3.1 mV; Figure S4b).

Equation 2, resulting from the dynamic expression of eq 1 with *z* = 1 (K^+^), , and *x* = *E*_app_(*t*), is used to fit the optical experimental curves and thus calculate the *A*_1_ and *A*_2_, *x*_0,1_, *k*_1_, and *n*_POT_ values for the system (Table S2).
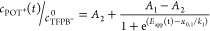
2

In all the cases, excellent fittings were exhibited (*R*^2^ = 0.9983–0.9995). We found that *A*_1_ = 1 and *A*_2_ = 0, as expected when only one sigmoidal part is displayed. Then, the average *k*_1_ = 0.079 V revealed a value for *n*_POT_ of 0.33, which agrees with previous predictions claiming that POT film oxidation manifests in a number of electrons lower than 1 and generally close to 0.35.^10,21^

Next, a model based on thermodynamic equilibrium assumptions was established to explain the empirically observed displacement of the peak with the logarithmic concentration of K^+^ by the Nernst equation. The ET at the ITO-POT interface is known to be a fast process that is not limited by doping through TFPB^–^. Hence, mass-transport limitation in the membrane or solution does not occur, meaning that the system can be described in a pure thin-layer regime.^10^ Then, *E*_app_ can be described by its distribution between the interconnected ET and IT:
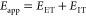
3

Yet, there is no empirical evidence about the weight, and so the energetic cost of each process (ET and IT) recognizes the total potential input provided to the system (*E*_app_). Although the realization of experimental observations for *E*_ET_ and/or *E*_IT_ is rather complex, some theoretical predictions pointed out the possibility of the ET process being the main contributor.^10^ Indeed, the average number of electrons related to POT oxidation (*n*_POT_) has been proposed as the main factor responsible for the voltammetric peak width.^10,21^

On the other hand, the generated current (*i*) upon the *E*_app_ is expected to be the same for all the points of the ITO-POT-membrane system, and consequently, it can be described for only one element or interface, for example, for POT oxidation as this is the process purely monitored with the optical measurements. Considering that the POT film is sufficiently thin (thickness is ca. 50 nm),^25^ the observed current is proportional to its oxidation rate (, the area of the electrode (*A*), and the thickness of the film (*d*_POT_), as follows:^26^
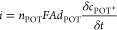
4

The POT oxidation rate can be predicted from eq 2, with *z*=2 , *A*_1_ = 0 and *A*_2_ = 1, *n*_POT_ = 0.33, and *k*_1_ = 0.079 V (as the average values found in the fittings; Table S2) as well as *x*_0,1_ = 0.802,0.827,0.880,0.906, and 0.949 V. Accordingly,  was calculated and later derived to obtain , predicting, hence, the voltammetric peak according to eq 4.

Following such a procedure, we can fully simulate both outputs from the spectroelectrochemical experiments, the optical curve, and the voltammetric peak, with good agreement with the experiments (Figure S5). Overall, while the values for *z* and *A* depend on the number of ITs, and *n*_POT_ (and hence *k_n_*) is fixed for the POT material, *x*_0,*n*_ is purely related to the IT, depending on the nature of the ion and its concentration in the sample solution. Certainly, in our previous studies, we demonstrated that *x*_0,*n*_ is related to the potential of the IT (*E*_IT_), fairly well coinciding with the peak potential of the CV.^20^ To provide adequate *x*_0,*n*_ values for the theoretical simulations, one may opt for two options: (i) *x*_0,*n*_ values referring to the standard potential of the IT (*E*_IT_^0^) or (ii) a semi-empirical approach, considering that *x*_0,*n*_ values are equal to the experimental peak potentials in the CVs, as we followed for the simulations in Figure S5.

To express the *E*_IT_ in terms of the *E*_IT_^0^, the Nernst equation can be used:
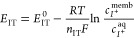
5where *c*_*I*^+^_^memb^ is the concentration of the ion analyte (*I^+^*) in the membrane being ideally fully bounded to the ionophore), *c*_*I*^+^_^aq^ is the concentration of *I^+^* in the aqueous solution, and *n*_IT_ is the number of electrons related to the IT process. Including the dynamic form of the mass balance for *c*_*I*^+^_^memb^ (eq 6), eq 7 is obtained, which relates the concentration of *I^+^* in the membrane with the gradual oxidation of POT^0^ to POT^+^.
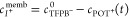
6
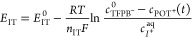
7

The mass balance deems that the concentration of *I^+^* in the membrane is equal to the TFPB^–^ concentration remaining after POT^+^ doping, which is only true if all the cationic positions in the membrane are initially filled by the ion analyte *I^+^*, i.e., under a thin-layer regime.

Finally, eq 7 can be inserted into eq 2, with *A*_1_ = 0, *A*_2_ = 1, and *k*_1_ = 0.079 V, for a numerical simulation of the POT oxidation rate, depending on the standard IT potential (eq 8). Such calculations are more complex than those involved in the semi-empirical approach, and hence, they were not adopted here. However, from eq 8, it was evident that the concentration of the ion in the aqueous solution (*c*_*I*^+^_^aq^) was expected to modify *E*_IT_ and, therefore, the potential for POT oxidation, which agreed with our experiments and is a valuable observation.
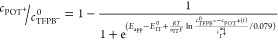
8

### Operational Mode 2. Cyclic Voltammetry at Micromolar K^+^ Levels: Mass-Transport Control

**Figure 3:**
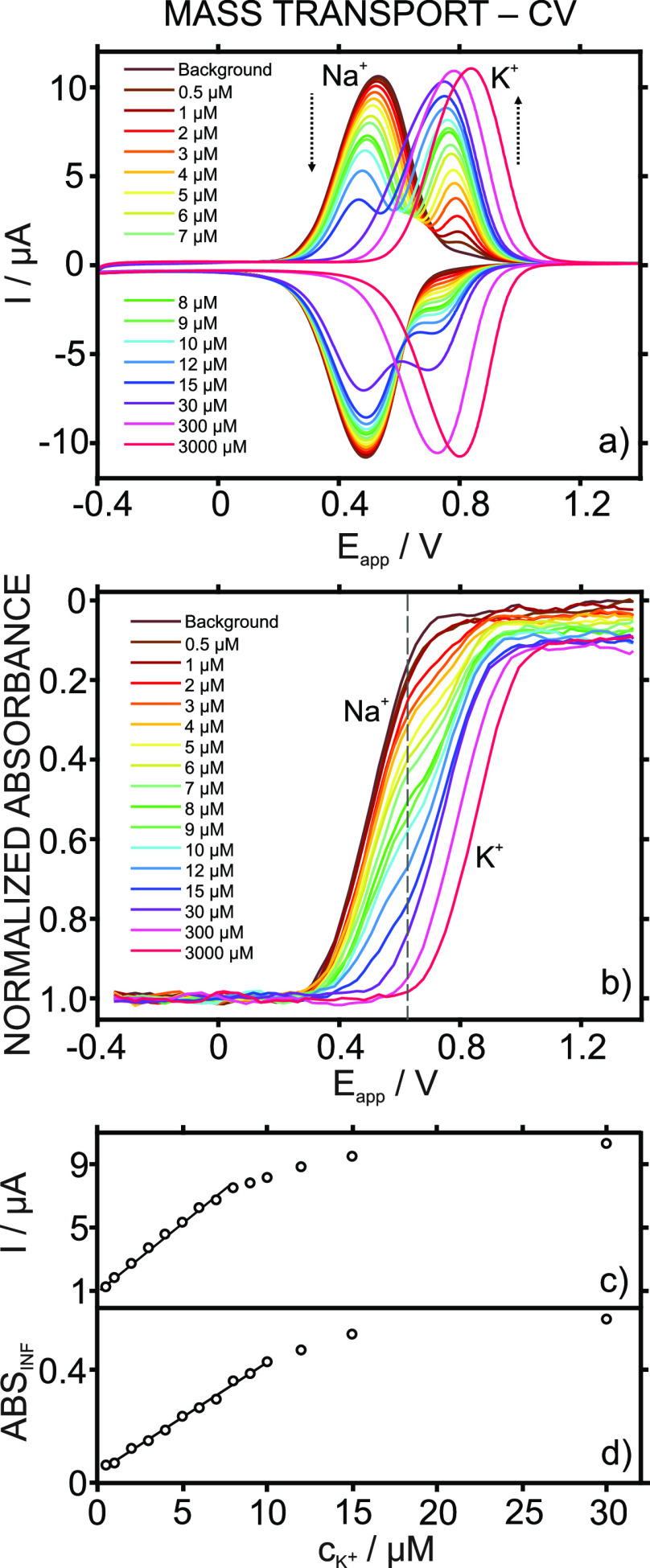
Experiments at the micromolar K^+^ concentration levels. (a) Cyclic voltammograms at increasing KCl concentrations with a 10 mM NaCl background. (b) Dynamic absorbance curves related to the anodic part of the voltammograms. Corresponding calibration curves using the (c) peak current and (d) normalized absorbance at the inflection point of the dynamic curve. Scan rate: 50 mV s^–1^. Wavelength: 450 nm.

Spectroelectrochemical experiments were carried out with the ITO-POT-membrane interrogated under CV at increasing KCl concentrations, ranging from 0.5 to 3000 μM, with a 10 mM NaCl background. The absorbance of the POT film was dynamically acquired at the same time as the CV. The CVs were run without providing any significant time for K^+^ to be accumulated in the membrane (30 s of stirring after each K^+^ addition). At micromolar K^+^ concentrations, it was expected that the IT process would be chiefly controlled by mass transport from the solution to the membrane, and with the mild stirring step, we aimed to avoid (or only minimally engage in) perturbing/promoting such transport. The CVs and the corresponding dynamic absorbances obtained in the described conditions are presented in Figure 3a and Figure 3b, respectively.

The CV for the lowest K^+^ concentration tested (0.5 μM) presented two peaks at 528.4 and 806.9 mV. The first peak was assigned to Na^+^ transfer further to comparison with the CV obtained in the background solution (only containing Na^+^). The second one was related to K^+^ transfer, as binding with the ionophore makes its expulsion from the membrane less favorable energetically, and thus, a higher potential is required. The first peak decreased, while the second one increased with the K^+^ concentration in the solution until a 30 μM K^+^ concentration was reached. From 30 μM K^+^ and above, only the K^+^ peak appeared and was found to shift to a more positive potential, indicating that a thin-layer regime was achieved from such a concentration (Figure 3a).

The total cationic sites available in the membrane (provided by the Na^+^TFPB^–^) are distributed between Na^+^ and K^+^ transfers from 0.5 to 30 μM K^+^ concentration in such a way that a higher K^+^ concentration in the sample solution promotes K^+^ accumulation in the membrane versus Na^+^. Notably, K^+^ accumulation is additionally facilitated by the presence of the ionophore, even though Na^+^ is at a higher concentration than K^+^ in the solution. From the 30 μM K^+^ concentration, the mass transport of K^+^ in the solution becomes a sufficiently fast process (under the established experimental conditions), and consequently, the membrane is filled by K^+^ instead of Na^+^, thus exhibiting the thin-layer behavior.

The behavior of the charge under the Na^+^ and K^+^ peaks agrees with the proposed description for the working mechanism. The charge of the initial Na^+^ peak (background solution) reflects the total exchange capacity of the membrane and was calculated to be 62.80 μC. Then, such a charge is distributed between the Na^+^ and the K^+^ peaks so that the sum of both charges is always equal (or very similar, 62.46 ± 0.56 μC) to that observed for the initial Na^+^ peak (see Figure S6). Moreover, the decrease and increase in the charge of the Na^+^ and K^+^ peaks were found to be linear. Regarding the increase in the current and charge for the K^+^ peak, while both parameters presented a concentration range with excellent linear correlation (Figure 3c and Figure S6, respectively), this was slightly wider in the case of the charge: 0.5–8 μM versus 0.5–10 μM for the current and charge, respectively. The linear fittings in these ranges were *I*_PEAK, K^+^_(μA) = 8.253 × 10^–1^*c*_K^+^_(μM) + 1.1246, *R*^2^ = 0.9937 and *Q*_K^+^_(μC) = 3.450 × *c*_K^+^_(μM) + 2.2114, *R*^2^ = 0.9943.

Dynamic absorbance curves at increasing K^+^ concentrations are depicted in Figure 3b. They presented shapes significantly different from those observed in the thin-layer CV experiments. The curve is divided into two sigmoidal-shaped parts (the separation of which is indicated by the dashed line in the figure) when the connected CV presents two well-defined peaks. The inflection points for each part coincide in turn with the peak potentials in the CV (see Table S3). Furthermore, the normalized absorbance at the inflection point (ABS_INF_) related to K^+^ transfer was found to linearly change with the K^+^ concentration in the solution from a 0.05 to 10 μM K^+^ concentration (Figure 3d): ABS_INF_ = 3.38 × 10^–2^*c*_K^+^_(μM) + 0.0322, *R*^2^ = 0.9965.

Thereafter, the potential at which the inflection point manifests shifted to more positive values with increasing K^+^ concentrations, coinciding with the thin-layer regime, and thus with the results previously observed for thin-layer CVs (Figure 3). Only one sigmoid appeared in such absorbance curves. The reproducibility of the absorbance responses was tested using a solution containing 5 μM KCl, with a 10 mM NaCl background (Figure S7). The dynamic absorbance curve was found to remain constant over consecutive CV scans and when checking three similar electrodes, with average inflection absorbance values of 0.206 ± 0.004 and 0.217 ± 0.009, respectively.

Of note, in the thin-layer CVs, no remarkable differences in either the linear range of response or the sensitivity (slope) were found when using the voltammetric peak potential or the inflection point potential (Figure 2). However, in the CVs under a diffusion-controlled mode, the fitting linear curve calculated from the dynamic absorbance exhibited a wider concentration range than that obtained with the voltammetric peak current, with the former closer to the linear range found for the peak charge (Figure S6). A possible explanation for this behavior is that the measurement of the POT absorbance is related to the charge formed in the film on oxidation, as demonstrated in our previous paper,^20^ and such a charge must fairly coincide with that under the voltammetric peak. Considering that some errors may be encountered in the numerical calculation of the charge under the voltammetric peak and given that the absorbance at the inflection point is a direct experimental measurement, it is more convenient to propose the use of this latter as the signal parameter in the calibration, especially considering the further analytical purpose of the methodology.

Equation 9, resulting from the dynamic expression of eq 1 with *z* = 2 (Na^+^ and K^+^), , and *x* = *E*_app_(*t*), was used to fit the experimental optical curves and calculate the *A*_1_, *A*_2_, *A*_3_, *x*_0,1_, *x*_0,2_, *k*_1_, *k*_2_, and *n*_POT_ values (Table S4).
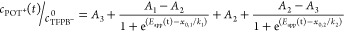
9

Excellent fittings were exhibited (*R*^2^ = 0.9983–0.9995) in all cases. We calculated that *A*_1_ = 0, *A*_3_ = 1, and *A*_2_ ranged from 1 to 0 as the K^+^ concentration increased. Meanwhile, the average values for *x*_0,1_ and *x*_0,2_ were 0.488 and 0.769 V, respectively, and the average value for *k_n_* was 0.060 V, thus revealing a value for *n*_POT_ of 0.43. The value for *A*_2_ was found to follow linearity, as determined by K^+^: *A*_2_ = – 5.34 × 10^–4^*c*_K^+^_(μM) + 0.94, *R*^2^ = 0.9928.

Modeling the system under mass transport limitation conditions is evidently more difficult than under thin-layer conditions. The mass balance should now consider that the available cationic positions, which are equal to the initial TFPB^–^ concentration in the membrane (*c*_TFPB^–^_^0^) minus the amount of oxidized POT^+^ (*c*_POT^+^_(*t*)), are distributed between both the ion in the background (*J^+^*) and the main ion (*I^+^*):
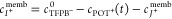
10

The initial concentrations of *I^+^* and *J^+^* in the membrane depend on the *I*^+^ concentration in the bulk solution, together with the conditions affecting its mass transport to the membrane phase. The initial concentration (or charge) of *I^+^* or *J^+^* in the membrane is expelled to the solution during the stripping step. Consequently, it is convenient to define the system by the amount of *I^+^* and *J^+^* initially present in the membrane. To that end, a continuity equation can be used to generally describe the propagation of concentration changes in *I^+^*, both in time and space in the aqueous solution, and considering, in turn, the diffusion coefficient of *I*^+^ (*D*_*I*^+^_^aq^):
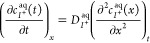
11

Hence, the aqueous solution can be approximated by a one-dimensional space–time grid (*x*, *t*). Moreover, we hypothesized that the space domain can be defined by the aqueous layer’s thickness (*d*_H or F_^aq^ ),as follows:
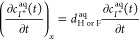
12with H denoting that the aqueous solution is a hydrodynamic layer under unstirred conditions and F denoting that the aqueous solution is a diffusion layer.^27−30^ In the absence of an accumulation step, as in the experiments in this section (Figure 3), H will apply to eq 12. Clearly, *d*_F_^aq^ will be lower than *d*_H_^aq^, and therefore, at the same *I^+^* concentration in the bulk solution, the rate of gain of *I^+^* in the membrane will be higher with the former. The rate of gain of *I^+^* in the membrane during the accumulation step must be equal to the rate of the loss of *I^+^* in the entire aqueous solution’s thickness (*d*_H_^aq^ or *d*_F_^aq^):
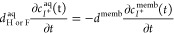
13

This can be considered in the expression for the mass balance (eq 10), and with the assumption that the aqueous solution is infinitely larger than the thin membrane domain (*d*_H or F_^aq^ ≫ *d*^memb^), we get the following:
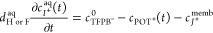
14

A similar reasoning can be applied to the *J^+^* gain in the solution during the accumulation step, with this, in turn, being equal to the rate of *I^+^* that the membrane gains or the aqueous solution loses
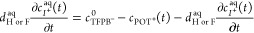
15

Then, solving eq 15 for *c*_POT^+^_(*t*) and discretizing with adequate boundary conditions and considering eq 4, it would be possible to calculate the current.

Though the purpose of the present work is not to provide such a solution to the current calculation, we consider it necessary to propose herein the fundamental equations that likely describe the system under study. The validity of the hypothesized expressions is expected to be confirmed in a follow-up piece of work more deeply dedicated to the theoretical description of the interconnected ET-IT mechanism. Nonetheless, in the work presented here, instead, a less complex semi-empirical approach based on eq 9 (*A*_1_ = 0, *A*_3_ = 1, and *A*_2_ calculated according to the found linearity; *x*_0,1_ = 0.488 V, *x*_0,2_ = 0.769 V, and *k_n_* = 0.43 V) was adopted to simulate the current profiles in the CVs under mass transport limitation conditions. By following such a procedure, we could adequately simulate both outputs from the spectroelectrochemical experiments, as presented in Figure S8. Again, the mathematical Sigmoidal–Boltzmann model showed a good performance for current prediction at increasing K^+^ concentrations. In principle, the averaged parameters for *A_n_* and *k*_1_*,* together with the *A*_2_ linearity discovered in this work, could be used to simulate the voltammograms for any IT at the micromolar level of concentration, just by iterating the values for *x*_0,1_ and *x*_0,2_.

### Operational Mode 3. Ion-Transfer Stripping Voltammetry at Nanomolar K^+^ Levels: Enhanced Diffusion Conditions

Spectroelectrochemical experiments were carried out with the ITO-POT-membrane interrogated under a striping anodic voltammetric protocol at increasing KCl concentrations, ranging from 10 to 3000 nM, with a 10 mM NaCl background. We implemented an initial accumulation step where the exchange of Na^+^ by K^+^ in the membrane was promoted. Thus, the entire electrochemical protocol was composed of an accumulation step (*E*_app_ = −0.2 V for 700 s, sample stirring at 400 rpm) followed by the application of an anodic linear sweep potential (from −0.2 to 1.4 V at a scan rate of 50 mV s^–1^). Details of the optimization of the accumulation/stripping protocol are provided in the Supporting Information (Figures S9–S11).

**Figure 4:**
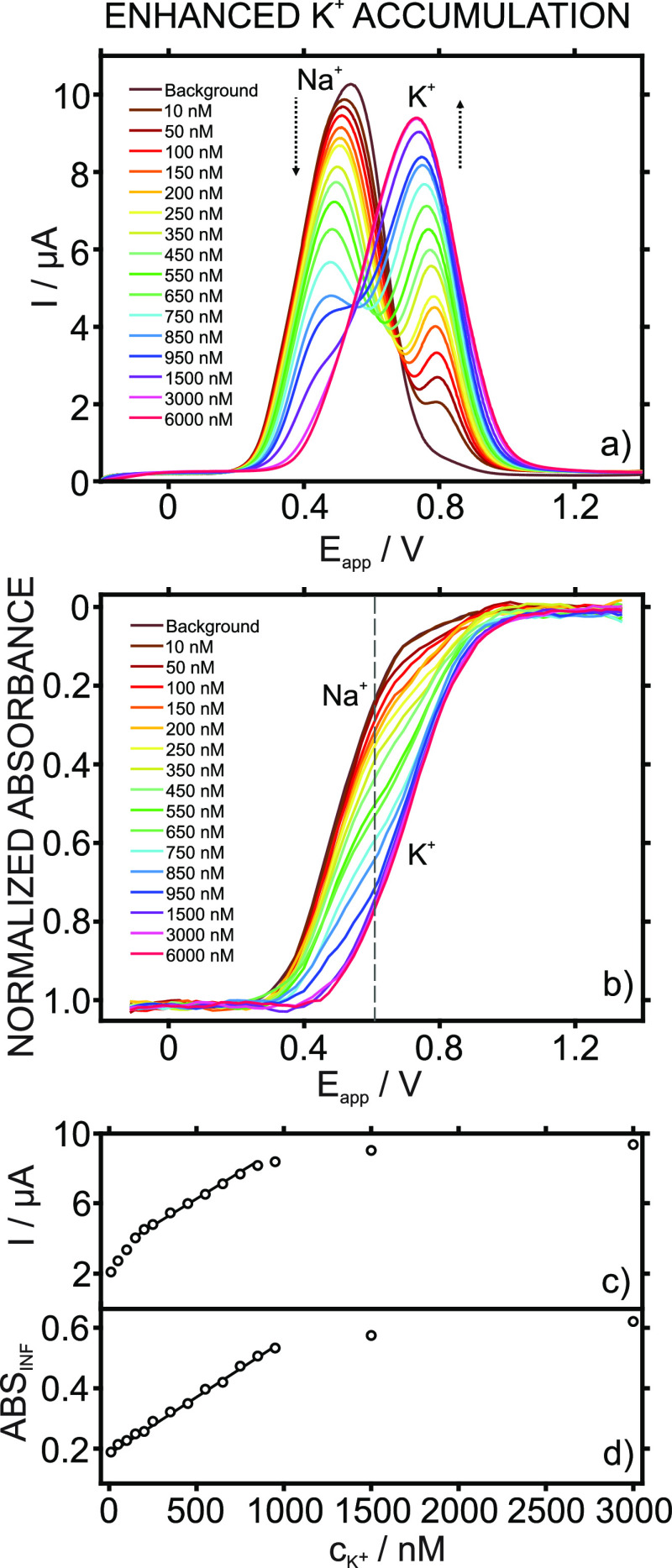
Experiments at the nanomolar K^+^ concentration levels. (a) Stripping voltammograms at increasing KCl concentrations with a 10 mM NaCl background. (b) Dynamic absorbance curves. Corresponding calibration curves using the (c) peak current and (d) normalized absorbance at the inflection point. Accumulation step (*E*_app_ = −0.2 V for 700 s while the sample solution is stirring at 400 rpm) followed by the application of an anodic linear sweep potential from −0.2 to 1.4 V (scan rate = 50 mV s^–1^). Wavelength: 450 nm.

The intentional accumulation step was expected to maximize the mass transport of K^+^ from the bulk solution to the membrane-solution interface, while the constant *E*_app_ ensured that the entire POT film was in the basal POT^0^ state. In the consecutive stripping step, the linear sweep potential resulted in the release of all the cation species from the membrane to the solution upon the oxidation of POT^0^ to POT^+^. Figure 4a displays the observed stripping voltammograms, and Figure 4b displays the corresponding dynamic absorbance traces. Even at a low concentration of 10 nM, the stripping voltammogram showed a peak for K^+^ at 792.8 mV. The peak was found to increase with increasing K^+^ concentrations in the sample solution, while the Na^+^ peak (528.1 mV) decreased. Moreover, the K^+^ peak current exhibited linearity with the K^+^ concentration from 150 to 850 nM (Figure 4c): *I*_PEAK, K^+^_(μA) = 5.80 × 10^–3^*c*_K^+^_(nM) + 3.332, *R*^2^ = 0.9947.

Our investigation of the charge under the peaks (Figure S12) revealed that the charge of the initial Na^+^ peak (62.53 μC) was well maintained as the total charge was distributed between the Na^+^ and K^+^ peaks (61.3 ± 0.40 μC). In addition, the charge of the K^+^ peak presented excellent linearity from a 10 to 950 nM concentration: *Q*_K^+^_(μC) = 4.43 × 10^–2^*c*_K^+^_(nM) + 6.992, *R*^2^ = 0.9937. This linear range of response was wider and began from a lower concentration (more than 10 times lower) than the linear range found for the peak current. Concerning the dynamic absorbance (Figure 4b), the curves appeared to be divided into two sigmoid-shaped parts (in which separation is indicated with a dashed line in the figure), well coinciding with the potential window for each voltammetric peak (Table S5 in the Supporting Information). On the other hand, the absorbance observed at the inflection point (ABS_INF_) of the K^+^ transfer part showed linearity with the K^+^ concentration from 10 to 950 nM (Figure 4d): ABS_INF_ = 3.385 × 10^–4^*c*_K^+^_(nM) + 0.0430, *R*^2^ = 0.9934. Importantly, the linear range was slightly wider than that observed for the charge under the voltammetric peak, which again confirmed the adequacy of the optical measurements for exploitation in the calibration graph in further analytical applications.

Equation 9 was used to fit the experimental optical curves and calculate the *A*_1_, *A*_2_, *A*_3_, *x*_0,1_, *x*_0,2_, *k*_1_, *k*_2_, and *n*_POT_ values (Table S6). An excellent correlation was obtained in all cases (*R*^2^ = 0.9969–0.9996). We found that *A*_1_ = 0, *A*_3_ = 1, and *A*_2_ ranged from 1 to 0 as the K^+^ concentration increased, while the average values for *x*_0,1_ and *x*_0,2_ were 0.495 and 0.772 V, respectively, and *k*_1_ = 0.060 V, which revealed a value for *n*_POT_ of 0.43. The value for *A*_2_ exhibited linearity with the K^+^: *A*_2_ = −7.59 × 10^–4^*c*_K_^+^(nM) + 0.94, *R*^2^ = 0.9927. These values were used to simulate the dynamic absorbance profiles and then to predict the voltammetric peak current according to eq 4. The results (Figure S13) were found to agree well with the experimental curves. Advantageously, the same parameters and the *A*_2_ linearity may be further used to predict the voltammograms related to any IT at the nanomolar level, just by iterating the values for *x*_0,1_ and *x*_0,2_, depending on the nature of the ion.

### Analytical Application of the Developed Spectroelectrochemical Methodology

**Figure 5:**
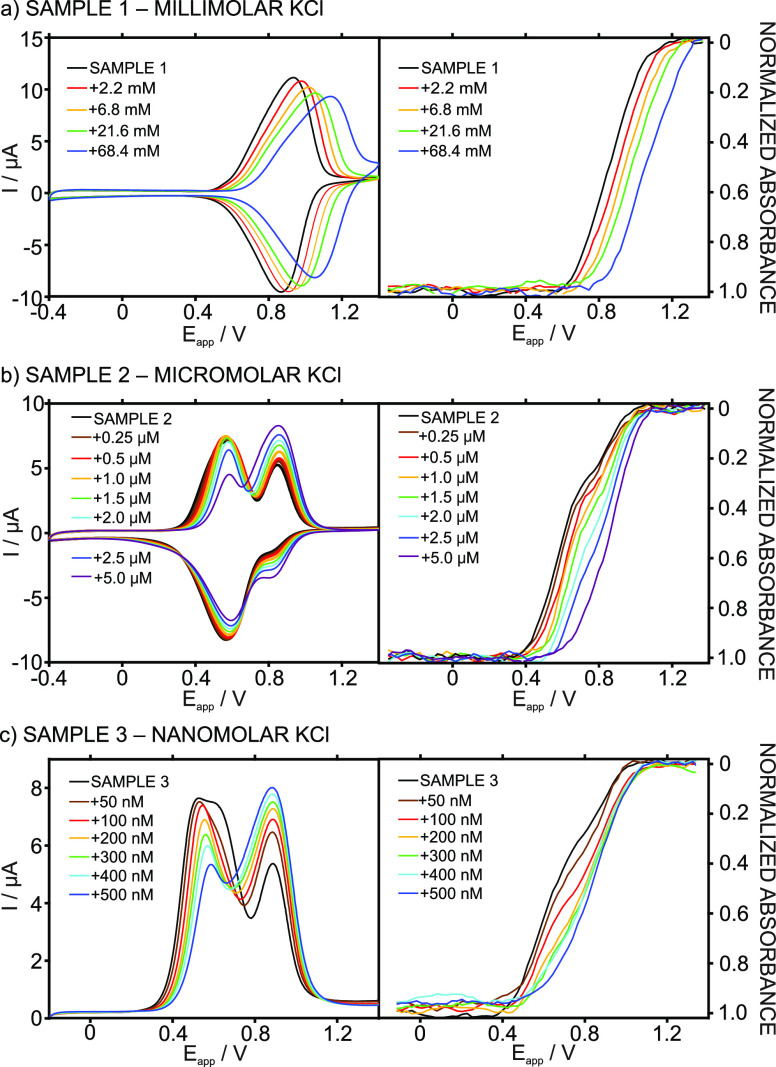
Cyclic voltammograms and dynamic absorbance curves for increasing concentrations of KCl in samples 1 (a), 2 (b), and 3 (c).

The analytical applicability of the developed ISE was evaluated by analyzing the K^+^ content, using the standard addition method, in six samples (see the Supporting Information): sample 1 (synthetic water with 1 mM KCl), sample 2 (synthetic water with 5 μM KCl), sample 3 (synthetic water with 300 nM KCl), sample 4 (distilled water), sample 5 (NaCl powder A with K^+^ traces), and sample 6 (NaCl powder B with K^+^ traces). The voltammograms and dynamic absorbance of the three synthetic water samples are presented in Figure 5, while those for samples 4–6 are depicted in Figure S14.

Sample 1 displayed only one peak at 937.8 V that shifted toward a more positive potential with subsequent KCl additions. Sample 2 presented two peaks (at 574.6 and 855.4 mV), the first of which (cations other than K^+^) decreased and the second (K^+^) of which increased with KCl additions. Sample 3 showed three peaks, the first two of which were at 527.8 and 593.8 mV (non-K^+^ cations) and the third of which (K^+^) was at 871.5 mV, with the two first peaks decreasing and the third one increasing with KCl additions. Samples 4–6 (Figure S14) presented two peaks, where the first (non-K^+^ cations) decreased and the second (K^+^) increased with KCl additions. All these results coincided with our expectations based on the K^+^ levels in each sample. Regarding the related optical curves, the total sigmoidal transient change was divided into a number of sigmoidal parts equal to the number of peaks, except for sample 3, where the first two peaks overlapped in only one sigmoidal part.

Table 1 presents the results of K^+^ concentration quantification in each sample using both the voltammogram (*E*_PEAK_ or *I*_PEAK_) and dynamic absorbance (*E*_INF_ or ABS_INF_), together with the error and recovery calculations. As can be observed, slightly better recoveries (closer to the 100%) were obtained when using the absorbance data, which confirmed our previous hypothesis that its use is preferable over the voltammetric data. Overall, the dataset largely demonstrated the analytical suitability of the developed spectroelectrochemical ISE for detecting the K^+^ concentration in different samples ranging from the nanomolar to the millimolar levels.

**Table 1:** Potassium Detection in Synthetic and Real Samples^a^

		absorbance		voltammogram	
sample	added K^+^	*c*_K^+^_	recovery (%)	*c*_K^+^_	recovery (%)
1	1 mM	0.90 mM^a^	90^e^	0.90 mM^c^	90^e^
2	5 μM	5.2 μM^b^	104^e^	4.8 μM^d^	96^e^
3	300 nM	252 nM^b^	84^e^	245 nM^d^	82^e^
4	–	184 nM^b^	–	215 nM^d^	–
	+50 nM	237 nM^b^	101^f^	302 nM^d^	114^f^
	+100 nM	289 nM^b^	101^f^	584 nM^d^	166^f^
5	–	642 nM^b^	–	653 nM^d^	–
	+50 nM	691 nM^b^	100^f^	896 nM^d^	127^f^
	+100 nM	750 nM^b^	101^f^	1021 nM^d^	108^f^
6	–	517 nM^b^	–	600 nM^d^	–
	+50 nM	585 nM^b^	103^f^	676 nM^d^	104^f^
	+100 nM	612 nM^b^	96^f^	980 nM^d^	140^f^

a For the calculations, the following parameters were used: *E*_INF_ (a), ABS_INF_ (b), *E*_PEAK_ (c), *I*_PEAK_ (d), difference with added K^+^ (e), and recovery (f).

## Conclusions

We have demonstrated that ISEs based on POT connected to ultrathin membranes can operate as spectroelectrochemical sensors in an unusually wide concentration range. In particular, we investigated the case of K^+^. Interrogating the corresponding ISE under different conditions (thin-layer CV, diffusion-controlled CV, or accumulation/stripping voltammetry), we found linear ranges of response in the nanomolar, micromolar, and millimolar K^+^ concentrations. While the three regimes fundamentally operate via an accumulation/expulsion mechanism, we ascertained that key to determining the different ranges of response is the accumulation part of that. This process, we found, dramatically depends on the amount of K^+^ present in the membrane, which in turn is ascribable to the mass transport of the ion from the bulk solution to the membrane. Beyond this, the mathematical Sigmoidal–Boltzmann model has been conveniently used to fit experimental data in searching for physicochemical parameters that may help calculate different spectroelectrochemical behaviors of an ISE. Moreover, equations enabling us to theoretically understand the working mechanism have been described to justify empirical observations of different regimes. Finally, the analytical applicability of the ISE has been illustrated with several samples of K^+^ at nanomolar, micromolar, and millimolar concentrations.

## Supporting Information Available

Experimental details, optimization of the conditions for operational mode 3, parameters for fittings and comparison with experimental values, real image of the setup, scheme of the mechanism, reproducibility, simulations, calculated charges, and data for the analysis of samples 4–6 (PDF)

## References

[ref1] Crespo G. A.; Bakker E. Dynamic electrochemistry with ionophore based ion-selective membranes. RSC Adv. 2013, 3, 25461–25474. 10.1039/C3RA43751E.

[ref2] Tatsumi S.; Omatsu T.; Maeda K.; Mousavi M. P. S.; Whitesides G. M.; Yoshida Y. An all-solid-state thin-layer laminated cell for calibration-free coulometric determination of K^+^. Electrochim. Acta 2022, 408, 139946 10.1016/j.electacta.2022.139946.

[ref3] Jansod S.; Ghahraman Afshar M.; Crespo G. A.; Bakker E. Alkalinization of thin layer samples with a selective proton sink membrane electrode for detecting carbonate by carbonate-selective electrodes. Anal. Chem. 2016, 88, 3444–3448. 10.1021/acs.analchem.6b00346. 26972891

[ref4] Xu K.; Crespo G. A.; Cuartero M. Subnanomolar detection of ions using thin voltammetric membranes with reduced Exchange capacity. Sens. Actuators, B 2020, 321, 128453 10.1016/j.snb.2020.128453.

[ref5] Cuartero M.; Crespo G. A.; Bakker E. Polyurethane ionophore-based thin layer membranes for voltammetric ion activity sensing. Anal. Chem. 2016, 88, 5649–5654. 10.1021/acs.analchem.6b01085. 27187779

[ref6] Han T.; Mattinen U.; Bobacka J. Improving the sensitivity of solid-contact ion-selective electrodes by using coulometric signal transduction. ACS Sens. 2019, 4, 900–906. 10.1021/acssensors.8b01649. 30844250 PMC6727616

[ref7] Węgrzyn K.; Kalisz J.; Stelmach E.; Maksymiuk K.; Michalska A. Emission Intensity Readout of Ion-Selective Electrodes Operating under an Electrochemical Trigger. Anal. Chem. 2021, 93, 10084–10089. 10.1021/acs.analchem.1c00857. 34264066 PMC8382224

[ref8] Kabagambe B.; Izadyar A.; Amemiya S. Stripping voltammetry of nanomolar potassium and ammonium ions using a valinomycin-doped double-polymer electrode. Anal. Chem. 2012, 84, 7979–7986. 10.1021/ac301773w. 22891987

[ref9] Kabagambe B.; Garada M. B.; Ishimatsu R.; Amemiya S. Subnanomolar detection limit of stripping voltammetric Ca^2+^-selective electrode: effects of analyte charge and sample contamination. Anal. Chem. 2014, 86, 7939–7946. 10.1021/ac501951m. 24992261

[ref10] Crespo G. A.; Cuartero M.; Bakker E. Thin layer ionophore-based membrane for multianalyte ion activity detection. Anal. Chem. 2015, 87, 7729–7737. 10.1021/acs.analchem.5b01459. 26161464

[ref11] Si P.; Bakker E. Thin layer electrochemical extraction of non-redoxactive cations with an anion-exchanging conducting polymer overlaid with a selective membrane. Chem. Commun. 2009, 5260–5262. 10.1039/b907893b. 19707639

[ref12] Cuartero M.; Chai L.; Zhang B.; De Marco R.; Crespo G. A. Ferrocene self assembled monolayer as a redox mediator for triggering ion transfer across nanometer-sized membranes. Electrochim. Acta 2019, 315, 84–93. 10.1016/j.electacta.2019.05.091.

[ref13] Yang Y.; Cuartero M.; Gonçales V. R.; Gooding J. J.; Bakker E. Light-Addressable Ion Sensing for Real-Time Monitoring of Extracellular Potassium. Am. Ethnol. 2018, 130, 17043–17047. 10.1002/ange.201811268. 30397985

[ref14] Jarolímová Z.; Bosson J.; Labrador G. M.; Lacour J.; Bakker E. Ion transfer voltammetry at thin films based on functionalized cationic [6] helicenes. Electroanalysis 2018, 30, 650–657. 10.1002/elan.201700669.

[ref15] Jansod S.; Wang L.; Cuartero M.; Bakker E. Electrochemical ion transfer mediated by a lipophilic Os (II)/Os (III) dinonyl bipyridyl probe incorporated in thin film membranes. Chem. Commun. 2017, 53, 10757–10760. 10.1039/C7CC05908F. 28884779

[ref16] Cuartero M.; Acres R. G.; Bradley J.; Jarolimova Z.; Wang L.; Bakker E.; Crespo G. A.; De Marco R. Electrochemical mechanism of ferrocene-based redox molecules in thin film membrane electrodes. Electrochim. Acta 2017, 238, 357–367. 10.1016/j.electacta.2017.04.047.

[ref17] Sohail M.; De Marco R.; Jarolímová Z.; Pawlak M.; Bakker E.; He N.; Latonen R.-M.; Lindfors T.; Bobacka J. Transportation and accumulation of redox active species at the buried interfaces of plasticized membrane electrodes. Langmuir 2015, 31, 10599–10609. 10.1021/acs.langmuir.5b01693. 26327251

[ref18] Cuartero M.; Crespo G. A.; Bakker E. Ionophore-based voltammetric ion activity sensing with thin layer membranes. Anal. Chem. 2016, 88, 1654–1660. 10.1021/acs.analchem.5b03611. 26712342

[ref19] Xu K.; Cuartero M.; Crespo G. A. Lowering the limit of detection of ion-selective membranes backside contacted with a film of poly (3-octylthiophene). Sens. Actuators, B 2019, 297, 126781 10.1016/j.snb.2019.126781.

[ref20] Liu Y.; Wiorek A.; Crespo G. A.; Cuartero M. Spectroelectrochemical Evidence of Interconnected Charge and Ion Transfer in Ultrathin Membranes Modulated by a Redox Conducting Polymer. Anal. Chem. 2020, 92, 14085–14093. 10.1021/acs.analchem.0c03124. 32972129 PMC7584340

[ref21] Liu Y.; Crespo G. A.; Cuartero M. Semi-empirical treatment of ionophore-assisted ion-transfers in ultrathin membranes coupled to a redox conducting polymer. Electrochim. Acta 2021, 388, 138634 10.1016/j.electacta.2021.138634.

[ref22] Ceresa A.; Sokalski T.; Pretsch E. Influence of key parameters on the lower detection limit and response function of solvent polymeric membrane ion-selective electrodes. J. Electroanal. Chem. 2001, 501, 70–76. 10.1016/S0022-0728(00)00488-5.

[ref23] Long R.; Bakker E. Optical determination of ionophore diffusion coefficients in plasticized poly (vinyl chloride) sensing films. Anal. Chim. Acta 2004, 511, 91–95. 10.1016/j.aca.2004.01.028.

[ref24] Yuan D.; Cuartero M.; Crespo G. A.; Bakker E. Voltammetric thin-layer ionophore-based films: part 1. Experimental evidence and numerical simulations. Anal. Chem. 2017, 89, 586–594. 10.1021/acs.analchem.6b03354. 27976858

[ref25] Cuartero M.; Acres R. G.; De Marco R.; Bakker E.; Crespo G. A. Electrochemical ion transfer with thin films of poly (3-octylthiophene). Anal. Chem. 2016, 88, 6939–6946. 10.1021/acs.analchem.6b01800. 27266678

[ref26] Osteryoung R. A.; Christie J. H. Theoretical treatment of pulsed voltammetric stripping at the thin film mercury electrode. Anal. Chem. 1974, 46, 351–355. 10.1021/ac60339a015.

[ref27] Molloy B.-J.; Tam K. Y.; Wood J. M.; Dryfe R. A. W. A hydrodynamic approach to the measurement of the permeability of small molecules across artificial membranes. Analyst 2008, 133, 655–659. 10.1039/b719634b. 18427688

[ref28] Madou M. J. Solid-State Physics, Fluidics, and Analytical Techniques in Micro-and Nanotechnology*;* CRC Press, 2011.

[ref29] Pohl P.; Saparov S. M.; Antonenko Y. N. The size of the unstirred layer as a function of the solute diffusion coefficient. Biophys. J. 1998, 75, 1403–1409. 10.1016/S0006-3495(98)74058-5. 9726941 PMC1299814

[ref30] Nikolic J.; Expósito E.; Iniesta J.; González-Garcia J.; Montiel V. Theoretical concepts and applications of a rotating disk electrode. J. Chem. Educ. 2000, 77, 1191 10.1021/ed077p1191.

