# Supplementary material for: Spectroelectrochemistry with Ultrathin Ion-Selective Membranes: Three Distinct Ranges for Analytical Sensing
Source: Anal Chem. 2022 Jun 10;94(25):9140–8. doi: 10.1021/acs.analchem.2c01584 (PMC9244873; doi:10.1021/acs.analchem.2c01584)
Supplement: Supplementary file 1 — ac2c01584_si_001.pdf [file ac2c01584_si_001.pdf]

Supporting Information for:

Spectroelectrochemistry with Ultra-Thin Ion-Selective Membranes: Three Distinct Ranges for Analytical Sensing

Yujie Liu, Gaston A. Crespo, Maria Cuartero*

Department of Chemistry, School of Engineering Sciences in Chemistry, Biotechnology and Health, KTH Royal Institute of Technology, Teknikringen 30, SE-100 44, Stockholm, Sweden.

*Corresponding author: mariacb@kth.se

Table of Contents

**Experimental**	Materials, instruments and preparations	S3
**Experimental**	Accumulation/Stripping protocol	S3
**Table S1**	Peaks information and absorbance transition information in Figure 2 (main manuscript)	S5
**Table S2**	Fitting Parameters calculated from Figure 2 (main manuscript)	S5
**Table S3**	Peaks information and absorbance transition information in Figure 3 (main manuscript)	S6
**Table S4**	Fitting Parameters calculated from Figure 3 (main manuscript)	S7
**Table S5**	Peaks information and absorbance transition information in Figure 4 (main manuscript)	S8
**Table S6**	Fitting Parameters calculated from Figure 4 (main manuscript)	S9
**Figure S1**	Picture of the spectroelectrochemical setup	S10
**Figure S2**	Scheme of the mechanism	S11
**Figure S3**	Real dynamic absorbance corresponding to Figure 2b (main manuscript)	S12
**Figure S4**	Reproducibility of dynamic absorbance for 1 mM KCl in 10 mM NaCl background	S13
**Figure S5**	Calculated voltammograms and dynamic absorbance for K^+ detection at millimolar level	S14
**Figure S6**	Charge vs. c_K corresponding to Figure 4 (main manuscript)	S15
**Figure S7**	Reproducibility of dynamic absorbance for 5 µM KCl in 10 mM NaCl background	S16
**Figure S8**	Calculated voltammograms and dynamic absorbance for K^+ detection at micromolar level	S17
**Figure S9**	Voltammograms and dynamic absorbance for 10 nM KCl using different accumulation time	S18
**Figure S10**	Voltammograms and dynamic absorbance for 10 nM KCl using different stirring speed	S19
**Figure S11**	Voltammograms and dynamic absorbance for 450 nM KCl using different stripping scan rate	S20
**Figure S12**	Charge vs. c_K corresponding to Figure 4 (main manuscript)	S21
**Figure S13**	Calculated voltammograms and dynamic absorbance for K^+ detection at nanomolar level	S22
**Figure S14**	Stripping voltammograms and dynamic absorbance for artificial samples	S23

Experimental

Reagents, materials and equipment. Aqueous solutions were prepared by dissolving the appropriate salts in deionized water (>18.2 MΩ).

Potassium chloride solution (0.001 M), 3-octylthiophene (97%, OT), lithium perchlorate (>98%, LiClO4), high molecular weight poly(vinyl chloride) (PVC), bis(2-ethylhexyl)sebacate (DOS), sodium tetrakis[3,5-bis-(trifluoromethyl)phenyl]borate (NaTFPB), potassium ionophore I (Valinomycin), sodium chloride (99.5% and 99.999%, NaCl), potassium chloride (99.5%, KCl), tetrahydrofuran (>99.9%, THF) and acetonitrile (anhydrous, >99.8%, ACN) were purchased from Sigma Aldrich. Absolute ethanol (99.5%, CH3CH2OH) and high purity grade NaCl was acquired in VWR.

Indium tin oxide (ITO) coated glass slides (25 mm × 25 mm × 1.1 mm, surface resistivity <10 Ω/sq, transmittance >83%) were sourced from Zhuhai Kaivo Optoelectronic Technology.

Cyclic voltammetry and linear voltammetry were performed by an Autolab PGSTAT204 potentiostat. An Autolab D/HAL light source (200-2500 nm, equipped with a software-controlled shutter) and a ferrule patch cable (M79L01, produced by Thorlabs) were used to generate illumination. Autolab Spectrophotometer UA (collecting wavelength range: 200–1100 nm), an optical cable from Dropsens (UV/VIS/NIR Optical fiber) and a lens were combined to record spectra. All instruments were controlled by Nova 2.1 software (supplied by Autolab). Calculations were accomplished in MATLAB_R2018b software.

Preparation of ITO-POT-membrane electrodes. First, the ITO electrode was cleaned with ethanol through ultra-sonification and rinsed with water. For the synthesis of POT, a solution comprising 0.1 M of both 3-octylthiophene and $LiClO_4$ in ACN was used. After degassing by purging nitrogen for 15 min, a POT film was electrochemically polymerized on the ITO surface by performing cyclic voltammetry (0–1.5 V, 100 mV/s, 2 scans) and then discharging at 0 V for 120 s. A Platinum electrode and a home-made Ag/AgCl wire were used as counter electrode and reference electrode respectively. Thereafter, the synthesized POT film was immersed in ACN and after in THF for 30 min and 10 s respectively and finally dried with air, to clean it from any residuals. A stock membrane cocktail was prepared by dissolving 15 mg of PVC, 30 mg of DOS, 1.8 mg of NaTFPB and 4.5 mg of potassium ionophore I in 500 µL of THF. The cocktail was diluted by mixing 50 µL of the stock solution with 150 µL of THF. A volume of 30 µL of the diluted membrane cocktail was deposited on the POT-ITO electrode by spin coating (1500 rpm, 60 s) using a 6808P spin coater provided by PI-KEM.

Potassium samples. A volume of 0.5 mL of ultra-pure NaCl solution (1 M, prepared by dissolving 99.999% purity NaCl powder in Milli-Q water) was mixed with 50 mL of the commercial 0.001 M KCl solution (Sigma) to provide a 1 mM standard KCl sample in NaCl background ***(Sample 1).*** Standard samples containing 5 µM and 300 nM KCl concentrations (***Sample 2*** and ***Sample 3*** respectively) were prepared by mixing 15 µL or 250 µL of the commercial 0.001 M KCl solution with 50 mL of 10 mM NaCl ultra-pure solution. A distilled water sample was collected from the lab tap supply. Ultra-pure NaCl powder was added into the distilled water sample to form a solution with 10 mM NaCl ***(Sample 4).*** Two powder samples (A: NaCl from Sigma and B: VWR, ***Sample 5*** and ***Sample 6***, respectively) were dissolved in milli-Q water to prepare two solutions of 10 mM NaCl concentration.

Optimization of the experimental conditions for the accumulation/stripping spectroelectrochemical protocol with enhanced accumulation (Operational mode 3). To optimize the accumulation/stripping protocol, the influence of the accumulation time and stirring speed on the electrode response were explored using a solution containing 10 nM K^+ concentration. The effect of scan rate was studied with a solution of 450 nM K^+ concentration.

Accumulation times ranging from 0 to 1500 s were evaluated while keeping constant the rest of parameters (stirring speed of 300 rpm, E_{app}=–0.2 V and scan rate of 50 mVs^{-1}). The results are shown in **Figure S7**. It was found that the voltammogram corresponding to an accumulation time as shorter as 30 s displays a similar shape as that observed at 0 s (i.e., no enhanced accumulation). When further expanded the accumulation time up to 700 s, the K^+ peak current in the CV keeps growing while the Na^+ peak current decreases. This indicates that a higher amount of K^+ is accumulated into the membrane, as the mass transport from the bulk solution to the membrane is promoted with increasing time. In the cases of an accumulation time larger than 700 s, any increase in the K^+ was less drastic. Regarding the dynamic absorbance curves, it is observed that more absorbance change occurs in the potassium region (second sigmoidal portion) when the accumulation process is enlarged from 30 s to 700 s. Notably, this change is less evident when accumulating for more than 700 s. Therefore, 700 s was selected as the optimized accumulation time.

The effect of the stirring speed of the sample solution during the accumulation step on the electrode response was studied from 100 to 800 rpm (**Figure S8**). A higher speed is not convenient in order to preserve the membrane integrity. A higher K^+ peak current was observed at increasing stirring speed, which is in principle ascribed to a diminishment in the thickness of the diffusion layer thickness, since K^+ mass transport becomes more efficient. Notably, there is a large difference in the peak currents display for 300 rpm and 400 rpm, after which the current profile did not show any significant change. Concerning the dynamic absorbance curves, the curve for 400 rpm demonstrates a larger absorbance change than the curves for 100, 200 and 300 rpm, while being rather similar as the curve for 500 rpm. As a result, a stirring speed of 400 rpm was selected as the optimal one.

Different scan rates in the stripping step, ranging from 25 to 100 mV s^{-1}, were also tested (**Figure S9**). When the scan rate was increased, a higher peak current was obtained for both Na^+ and K^+. However, the ratio between the two peak currents slightly differs, as K^+ peak is slightly favored over the Na^+ one. Inspecting the optical curves, two distinct transitions, corresponding to Na^+ transfer and K^+ transfer, can be clearly recognized in the curves for 10 mV/s, 25 mV/s and 50 mV/s. When using an even higher scan rate, the boundary between the two transitions is hard to be distinguished, because the time for collecting optical signal seems to be insufficient to provide an acceptable resolution. Accordingly, a scan rate of 50 mV s^{-1} was selected as the optimal one.

Tables

Table S1. Parameters for the experimental voltammograms and dynamic normalized absorbance at increasing concentration of KCl at millimolar level in 10 mM NaCl background.

c_{K^+} / mM	Voltammograms		Absorbance transition	
	from–to, mV	E_{PEAK,K^+} / mV	from–to, mV	E_{INF} / mV
0.5	423.4–1139.8	856.9	491.9–1077.5	802.0
1	438.0–1158.2	884.5	462.6–1145.2	827.3
5	462.3–1194.0	942.7	486.9–1181.7	880.1
10	511.3–1243.3	973.5	584.6–1195.8	906.0
32	560.2–1255.0	1015.0	599.2–1233.0	949.0

Table S2. Parameters for the calculated voltammograms and dynamic normalized absorbance at increasing concentration of KCl at millimolar level in 10 mM NaCl background. The average values are then used to simulate the absorbance curves and the voltammograms provided in **Figure S5**. SD=standard deviation.

c_{K^+} / mM	A_n	$x_{0,1}$ (V)	k (V)	n_{POT}	R^2
0.5	A_1: 0<br>A_2: 1	0.802	0.077	*0.333*	*0.9983*
1	A_1: 0<br>A_2: 1	0.827	0.077	*0.333*	*0.9995*
5	A_1: 0<br>A_2: 1	0.880	0.079	*0.325*	*0.9991*
10	A_1: 0<br>A_2: 1	0.906	0.079	*0.325*	*0.9987*
32	A_1: 0<br>A_2: 1	0.949	0.081	*0.317*	*0.9983*
**Average±SD**	A_1: 0<br>A_2: 1	–	*0.079*±0.002	*0.327*±0.006	*0.9988*±0.0004

Table S3. Parameters for the experimental voltammograms and normalized dynamic absorbance at increasing concentration of KCl at micromolar level in 10 mM NaCl background.

c_{K^+} / μM	Voltammograms		Absorbance transition	
	E_{PEAK,Na^+} / mV	E_{PEAK,K^+} / mV	E_{INF,Na^+} / mV	E_{INF,K^+} / mV
0	530.9	–	507.0	–
0.5	528.4	801.4	503.2	789.3
1	526.0	793.8	500.4	785.5
2	523.5	793.8	496.3	785.0
3	518.7	786.7	493.8	780.3
4	516.2	781.3	491.5	779.0
5	511.3	778.8	487.7	774.7
6	506.3	772.4	484.5	770.8
7	501.5	769.3	483.0	764.5
8	496.6	764.2	479.9	760.6
9	494.2	764.2	477.0	760.2
10	486.8	761.7	475.3	758.1
12	477.8	754.4	471.4	750.0
15	465.3	749.2	468.1	747.2
30	–	745.8	–	743.6
300	–	784.3	–	798.4
3000	–	840.5	–	854.4

Table S4. Parameters for the calculated voltammograms and dynamic normalized absorbance at increasing concentration of KCl at micromolar level in 10 mM NaCl background. The average values are then used to simulate the absorbance curves and the voltammograms provided in **Figure S8**. SD=standard deviation.

c_{K^+} / μM	A_n	$x_{0,n}$ (V)	k_n (V)	n_{POT}	R^2
0	$A_1 = 0$<br>$A_2 = 1$<br>$A_3 = 1$	$x_{0,1} = 0.5070$	$k_1 = 0.071$	$n_1 = 0.362$	0.9990
0.5	$A_1 = 0$<br>$A_2 = 0.9037$<br>$A_3 = 1$	$x_{0,1} = 0.5032$<br>$x_{0,2} = 0.7893$	$k_1 = 0.062$<br>$k_2 = 0.050$	$n_1 = 0.414$<br>$n_2 = 0.514$	0.9988
1	$A_1 = 0$<br>$A_2 = 0.8914$<br>$A_3 = 1$	$x_{0,1} = 0.5004$<br>$x_{0,2} = 0.7855$	$k_1 = 0.062$<br>$k_2 = 0.080$	$n_1 = 0.414$<br>$n_2 = 0.321$	0.9994
2	$A_1 = 0$<br>$A_2 = 0.8000$<br>$A_3 = 1$	$x_{0,1} = 0.4963$<br>$x_{0,2} = 0.785$	$k_1 = 0.053$<br>$k_2 = 0.054$	$n_1 = 0.484$<br>$n_2 = 0.476$	0.9986
3	$A_1 = 0$<br>$A_2 = 0.7601$<br>$A_3 = 1$	$x_{0,1} = 0.4938$<br>$x_{0,2} = 0.7803$	$k_1 = 0.050$<br>$k_2 = 0.048$	$n_1 = 0.514$<br>$n_2 = 0.535$	0.9990
4	$A_1 = 0$<br>$A_2 = 0.7393$<br>$A_3 = 1$	$x_{0,1} = 0.4915$<br>$x_{0,2} = 0.779$	$k_1 = 0.055$<br>$k_2 = 0.070$	$n_1 = 0.467$<br>$n_2 = 0.414$	0.9995
5	$A_1 = 0$<br>$A_2 = 0.6864$<br>$A_3 = 1$	$x_{0,1} = 0.4877$<br>$x_{0,2} = 0.7747$	$k_1 = 0.055$<br>$k_2 = 0.070$	$n_1 = 0.467$<br>$n_2 = 0.367$	0.9989
6	$A_1 = 0$<br>$A_2 = 0.6446$<br>$A_3 = 1$	$x_{0,1} = 0.4845$<br>$x_{0,2} = 0.7708$	$k_1 = 0.051$<br>$k_2 = 0.056$	$n_1 = 0.503$<br>$n_2 = 0.459$	0.9988
7	$A_1 = 0$<br>$A_2 = 0.5764$<br>$A_3 = 1$	$x_{0,1} = 0.4830$<br>$x_{0,2} = 0.7645$	$k_1 = 0.051$<br>$k_2 = 0.077$	$n_1 = 0.503$<br>$n_2 = 0.333$	0.9976
8	$A_1 = 0$<br>$A_2 = 0.5040$<br>$A_3 = 1$	$x_{0,1} = 0.4799$<br>$x_{0,2} = 0.7606$	$k_1 = 0.048$<br>$k_2 = 0.073$	$n_1 = 0.535$<br>$n_2 = 0.352$	0.9951
9	$A_1 = 0$<br>$A_2 = 0.4569$<br>$A_3 = 1$	$x_{0,1} = 0.477$<br>$x_{0,2} = 0.7602$	$k_1 = 0.052$<br>$k_2 = 0.069$	$n_1 = 0.494$<br>$n_2 = 0.372$	0.9967
10	$A_1 = 0$<br>$A_2 = 0.3957$<br>$A_3 = 1$	$x_{0,1} = 0.4753$<br>$x_{0,2} = 0.7581$	$k_1 = 0.045$<br>$k_2 = 0.074$	$n_1 = 0.571$<br>$n_2 = 0.347$	0.9940
12	$A_1 = 0$<br>$A_2 = 0.2812$<br>$A_3 = 1$	$x_{0,1} = 0.4714$<br>$x_{0,2} = 0.7500$	$k_1 = 0.044$<br>$k_2 = 0.073$	$n_1 = 0.584$<br>$n_2 = 0.352$	0.9946
15	$A_1 = 0$<br>$A_2 = 1870$<br>$A_3 = 1$	$x_{0,1} = 0.4781$<br>$x_{0,2} = 0.7472$	$k_1 = 0.043$<br>$k_2 = 0.073$	$n_1 = 0.597$<br>$n_2 = 0.352$	0.9913
30	$A_1 = 0$<br>$A_2 = 0$<br>$A_3 = 1$	$x_{0,2} = 0.7436$	$k_1 = 0.083$	$n_2 = 0.309$	0.9941
300	$A_1 = 0$<br>$A_2 = 0$<br>$A_3 = 1$	$x_{0,2} = 0.7984$	$k_1 = 0.076$	$n_2 = 0.338$	0.9905
3000	$A_1 = 0$<br>$A_2 = 1$<br>$A_3 = 1$	$x_{0,2} = 0.8544$	$k_1 = 0.074$	$n_2 = 0.347$	0.9955
Average±SD	–	$x_{0,1} = 0.4878 \pm 0.0108$<br>$x_{0,2} = 0.0137 \pm 0.0137$	0.060±0.012*	0.437±0.086*	0.9966±0.0028*

*30, 300 and 3000 μM are not included.

Table S5. Parameters for the experimental voltammograms and normalized dynamic absorbance at increasing concentrations of KCl at nanomolar levels in 10 mM NaCl background.

c_{K^+} / nM	Voltammograms		Absorbance transition	
	E_{PEAK,Na^+} / mV	E_{PEAK,K^+} / mV	E_{INF,Na^+} / mV	E_{INF,K^+} / mV
0	528.1	–	504.1	–
10	518.3	792.8	501.0	800.0
50	513.5	795.9	517.5	796.8
100	511.1	793.2	512.0	794.0
150	511.0	791.0	509.2	788.5
200	506.1	786.1	509.2	782.9
250	503.6	783.7	506.5	782.9
350	498.8	777.6	503.7	780.2
450	496.3	773.9	489.9	771.9
550	491.5	770.0	487.1	766.4
650	484.1	763.5	476.0	760.8
750	477.6	754.1	473.3	755.3
850	475.0	748.9	476.0	749.9
950	479.8	748.0	481.6	747.0
1500	464.9	740.8	470.5	735.9
3000	–	735.7	–	727.6
6000	–	733.4	–	730.0

Table S6. Parameters for the calculated voltammograms and dynamic normalized absorbance at increasing concentration of KCl at nanomolar level in 10 mM NaCl background. The average values are then used to simulate the absorbance curves and the voltammograms provided in **Figure S13**. SD=standard deviation.

c_{K^+} / nM	A_n	$x_{0,n}$ (V)	k_n (V)	n_{POT}	R^2
0	$A_1 = 0,$ $A_2 = 1$ $A_3 = 1$	$x_{0,1} = 0.5041$	$k_1 = 0.076$	$n_1 = 0.338$	0.9991
10	$A_1 = 0$ $A_2 = 0.9516$ $A_3 = 1$	$x_{0,1} = 0.5010$ $x_{0,2} = 0.8000$	$k_1 = 0.068$ $k_2 = 0.070$	$n_1 = 0.378$ $n_2 = 0.367$	0.999
50	$A_1 = 0$ $A_2 = 0.9192$ $A_3 = 1$	$x_{0,1} = 0.5175$ $x_{0,2} = 0.7968$	$k_1 = 0.066$ $k_2 = 0.064$	$n_1 = 0.389$ $n_2 = 0.401$	0.9976
100	$A_1 = 0$ $A_2 = 0.886$ $A_3 = 1$	$x_{0,1} = 0.5120$ $x_{0,2} = 0.7940$	$k_1 = 0.067$ $k_2 = 0.067$	$n_1 = 0.383$ $n_2 = 0.383$	0.9983
150	$A_1 = 0$ $A_2 = 0.7899$ $A_3 = 1$	$x_{0,1} = 0.5092$ $x_{0,2} = 0.7885$	$k_1 = 0.059$ $k_2 = 0.056$	$n_1 = 0.435$ $n_2 = 0.459$	0.9976
200	$A_1 = 0$ $A_2 = 0.7714$ $A_3 = 1$	$x_{0,1} = 0.5092$ $x_{0,2} = 0.7829$	$k_1 = 0.062$ $k_2 = 0.058$	$n_1 = 0.414$ $n_2 = 0.443$	0.9969
250	$A_1 = 0$ $A_2 = 0.7271$ $A_3 = 1$	$x_{0,1} = 0.5065$ $x_{0,2} = 0.7829$	$k_1 = 0.057$ $k_2 = 0.057$	$n_1 = 0.450$ $n_2 = 0.450$	0.9974
350	$A_1 = 0$ $A_2 = 0.6728$ $A_3 = 1$	$x_{0,1} = 0.5037$ $x_{0,2} = 0.7802$	$k_1 = 0.059$ $k_2 = 0.058$	$n_1 = 0.435$ $n_2 = 0.443$	0.9972
450	$A_1 = 0$ $A_2 = 0.5958$ $A_3 = 1$	$x_{0,1} = 0.4899$ $x_{0,2} = 0.7719$	$k_1 = 0.053$ $k_2 = 0.062$	$n_1 = 0.484$ $n_2 = 0.414$	0.9986
550	$A_1 = 0$ $A_2 = 0.5027$ $A_3 = 1$	$x_{0,1} = 0.4871$ $x_{0,2} = 0.7664$	$k_1 = 0.048$ $k_2 = 0.064$	$n_1 = 0.535$ $n_2 = 0.401$	0.9987
650	$A_1 = 0$ $A_2 = 0.4546$ $A_3 = 1$	$x_{0,1} = 0.4760$ $x_{0,2} = 0.7608$	$k_1 = 0.054$ $k_2 = 0.063$	$n_1 = 0.476$ $n_2 = 0.408$	0.9992
750	$A_1 = 0$ $A_2 = 0.366$ $A_3 = 1$	$x_{0,1} = 0.4733$ $x_{0,2} = 0.7553$	$k_1 = 0.041$ $k_2 = 0.063$	$n_1 = 0.626$ $n_2 = 0.408$	0.9996
850	$A_1 = 0$ $A_2 = 0.3191$ $A_3 = 1$	$x_{0,1} = 0.4760$ $x_{0,2} = 0.7498$	$k_1 = 0.049$ $k_2 = 0.064$	$n_1 = 0.524$ $n_2 = 0.401$	0.9996
950	$A_1 = 0$ $A_2 = 0.211$ $A_3 = 1$	$x_{0,1} = 0.4816$ $x_{0,2} = 0.7470$	$k_1 = 0.044$ $k_2 = 0.069$	$n_1 = 0.584$ $n_2 = 0.372$	0.9991
1500	$A_1 = 0$ $A_2 = 0.0771$ $A_3 = 1$	$x_{0,1} = 0.4705$ $x_{0,2} = 0.7359$	$k_1 = 0.040$ $k_2 = 0.070$	$n_1 = 0.642$ $n_2 = 0.367$	0.9964
3000	$A_1 = 0$ $A_2 = 0$ $A_3 = 1$	$x_{0,2} = 0.7276$	$k_2 = 0.086$	$n_2 = 0.299$	0.9961
6000	$A_1 = 0$ $A_2 = 0$ $A_3 = 1$	$x_{0,2} = 0.7300$	$k_2 = 0.084$	$n_2 = 0.306$	0.9973
Average±SD	–	$x_{0,1} = 0.4945 \pm 0.0154$ $x_{0,2} = 0.7322 \pm 0.1013$	0.060±0.009*	0.433±0.080*	0.9981±0.0011*

*3000 and 6000 nM are not included.

Figures

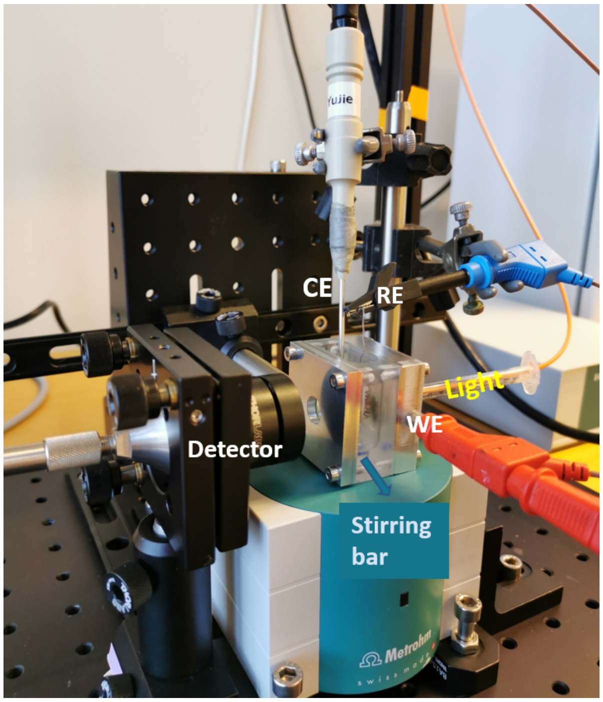

Figure S1. Picture of the spectroelectrochemical setup based on the cell presented in Figure 1 in the main manuscript. WE: working electrode, CE: counter electrode, RE: reference electrode.

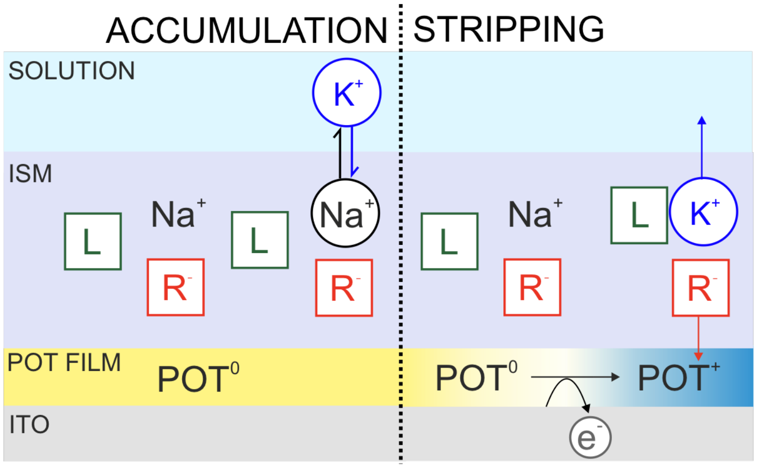

Figure S2. Schematic representation of the accumulation/stripping principle in the ITO-POT-membrane electrode. ISM=Ion-selective membrane. L=ionophore. R^-=$TFPB^-$. The oxidation form POT^0 to POT^+ manifests in a change of the optical signal at 450 nm.

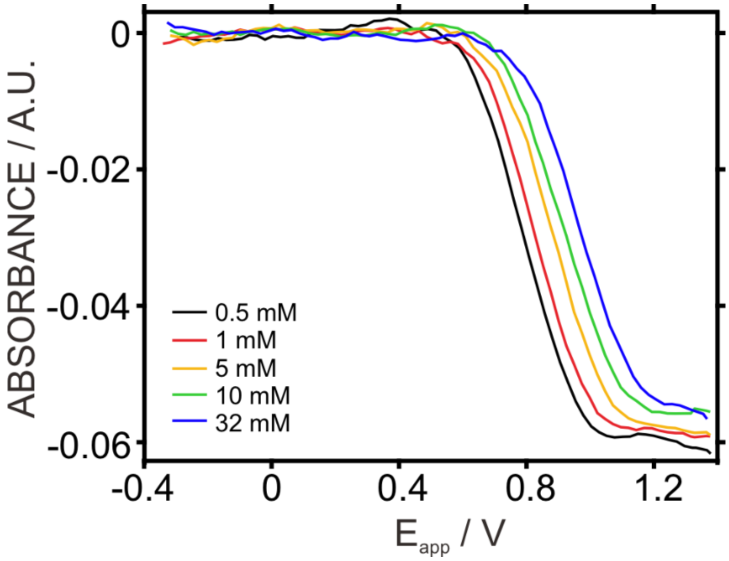

Figure S3. Untreated dynamic absorbance curves related to **Figure 2b** in the main manuscript. Scan rate: 50 mV s^{-1}. Wavelength: 450 nm.

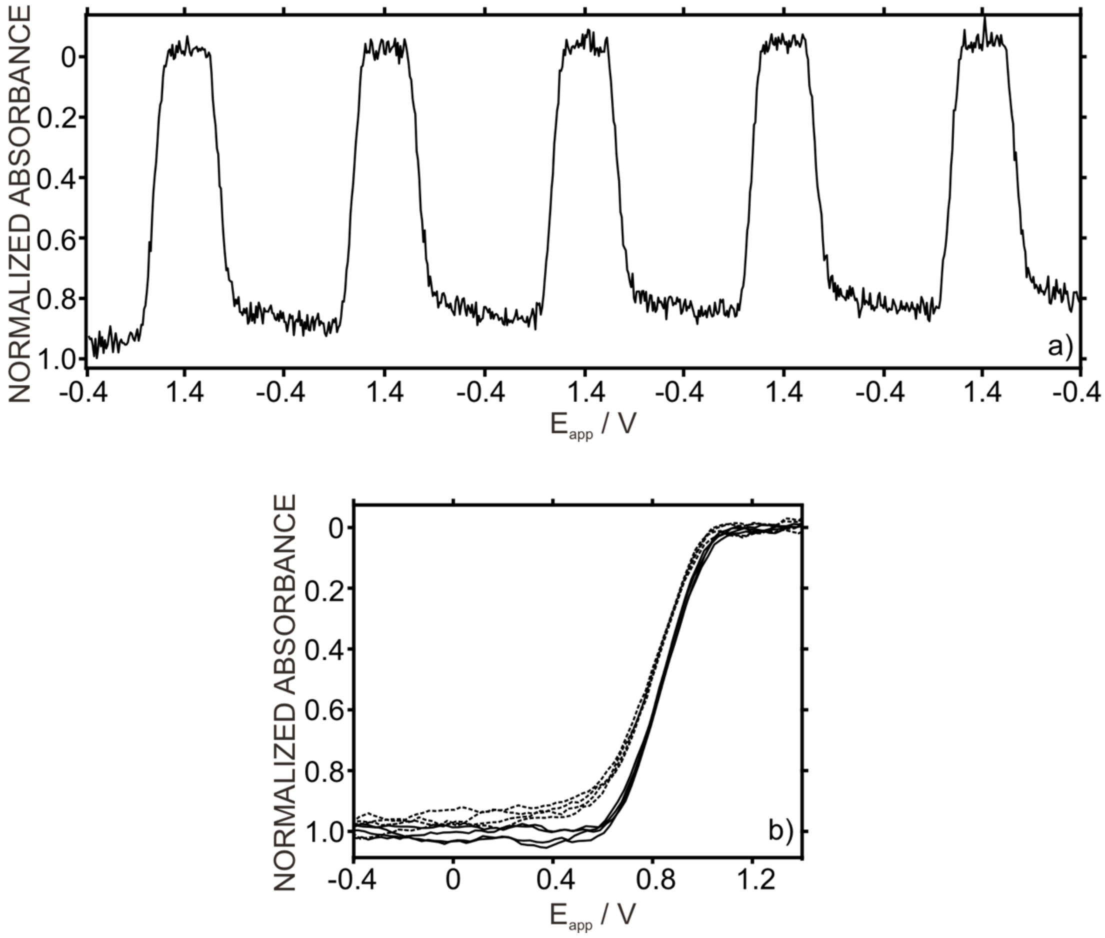

Figure S4. (a) Dynamic absorbance for 1 mM KCl in 10 mM NaCl background at five consecutive cyclic voltammetry scans. Scan rate: 50 mV s^{-1}. (b) The same data but grouped per voltammetry cycle. Solid line=anodic scan. Dashed line=cathodic scan.

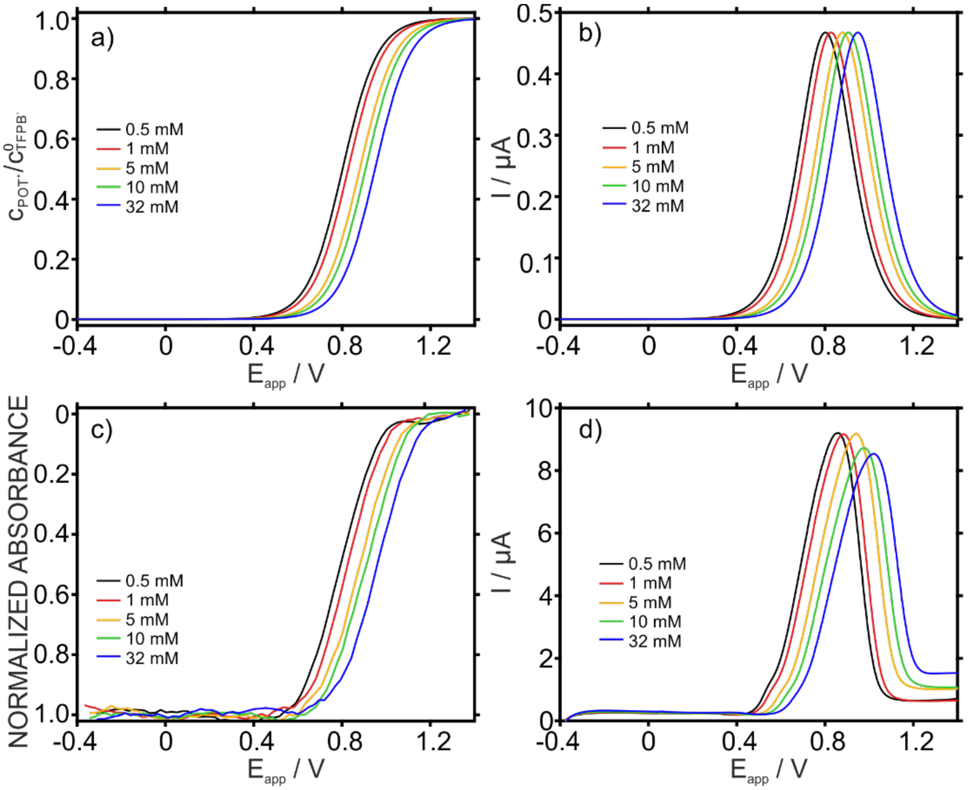

Figure S5. For increasing KCl concentrations at the millimolar level in 10 mM NaCl background solution: (a) calculated $\frac{c_{POT^+}}{c^0_{TFPB^-}}$ profiles, (b) calculated voltammograms, (c) experimental dynamic absorbance and (d) experimental voltammograms of the POT film. Scan rate: 50 mV s^{-1}. Notably, the experimental results (c and d) are added for comparative purposes.

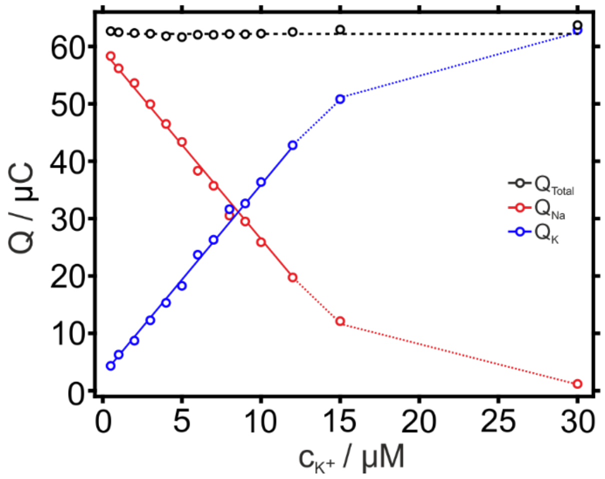

Figure S6. Plot of the peak charge corresponding to the Na^+ and K^+ voltammetric peaks in the experiment in **Figure 3** (main manuscript). The sum of both charges versus the K^+ concentration is also presented. Linear fittings: $Q_{Na^+}(\mu C) = -3.425 \times c_K(\mu M) + 59.892, R^2 = 0.9960$ and $Q_{K^+}(\mu C) = 3.415 \times c_K(\mu M) + 2.342, R^2 = 0.9956$.

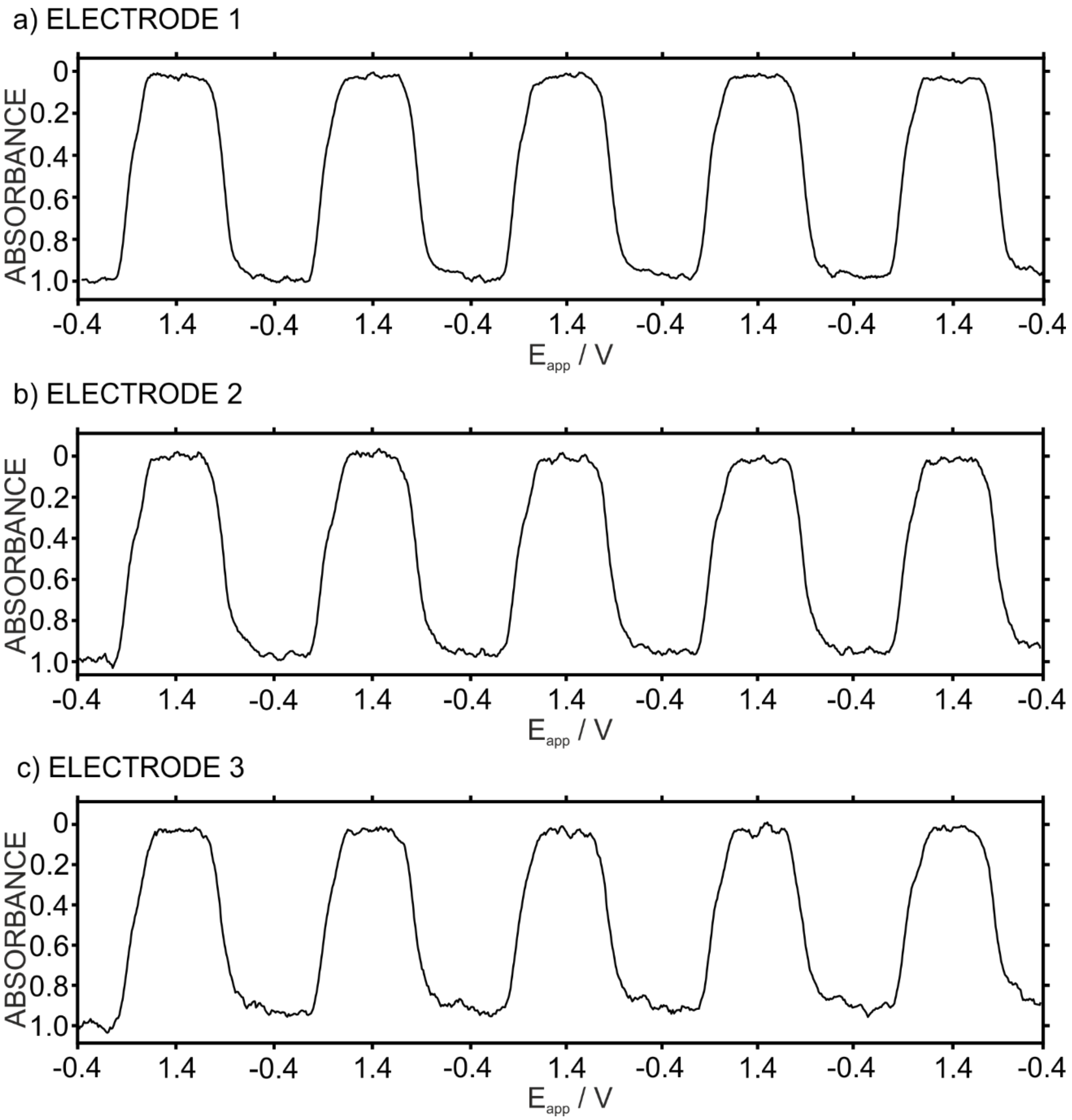

Figure S7. Normalized dynamic absorbance of three electrodes for 5 μM KCl in 10 mM NaCl background at five consecutive cyclic voltammetry scans. Scan rate: 50 mV s^{-1}.

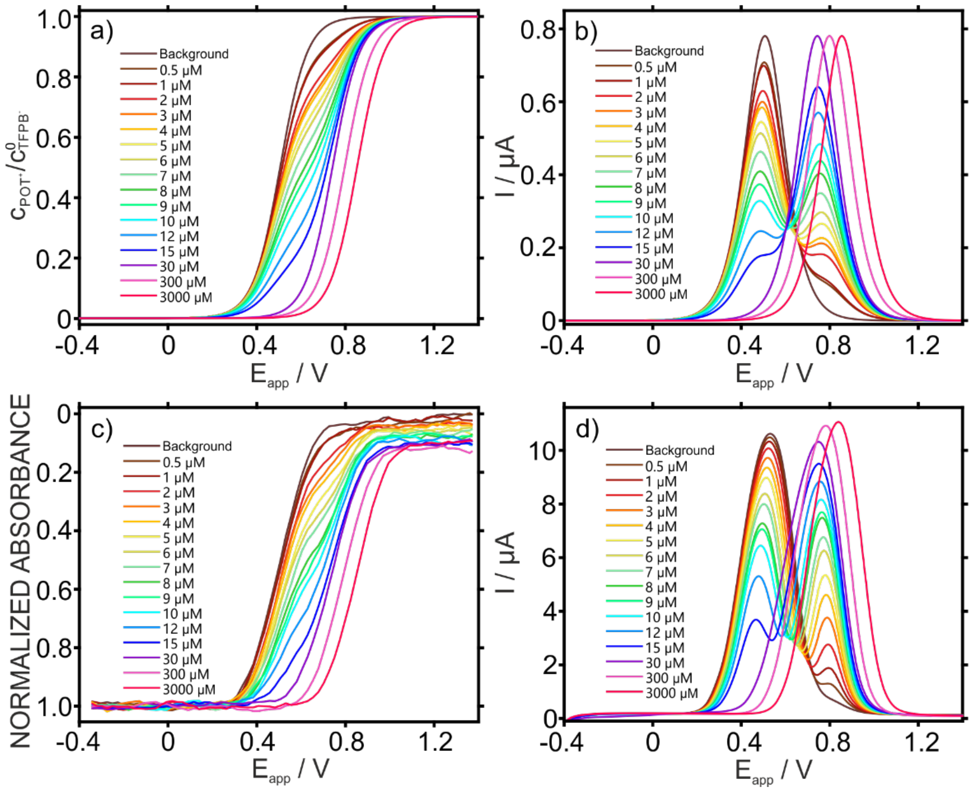

Figure S8. For increasing KCl concentrations at the micromolar levels in 10 mM NaCl background solution: (a) calculated $\frac{c_{POT^+}}{c^0_{TFPB^-}}$ profiles, (b) calculated voltammograms, (c) experimental dynamic absorbance and (d) experimental voltammograms of the POT film. Scan rate: 50 mV s^{-1}. Notably, the experimental results (c and d) are added for comparative purposes.

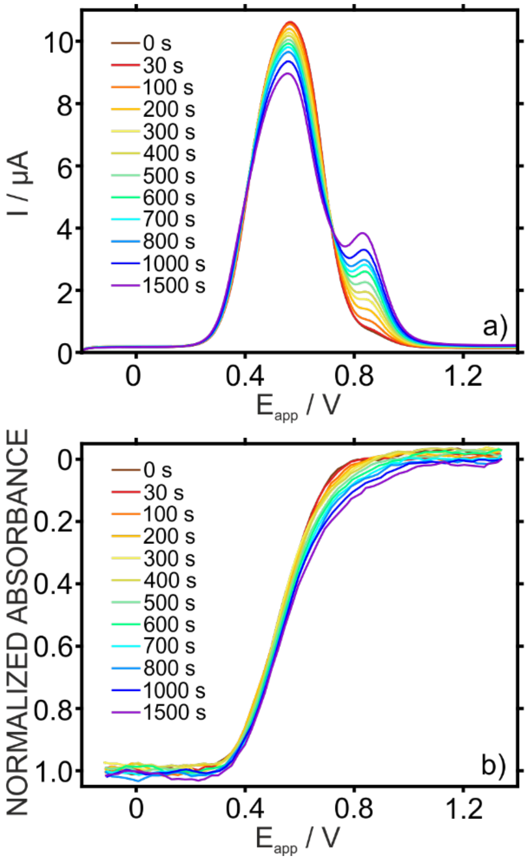

Figure S9. Accumulation/stripping protocol optimization: (a) Stripping voltammograms and (b) the associated dynamic absorbance curves for 10 nM KCl in 10 mM NaCl solution at increasing accumulation time of 0, 30, 100, 200, 300, 400, 500, 600, 700, 800, 1000 and 1500 s. Electrochemical protocol: E_{app}=–0.2 V during the accumulation with the stirring speed of 300 rpm, linear sweep stripping from –0.2 to 1.4 V, scan rate of 50 mV s^{-1}.

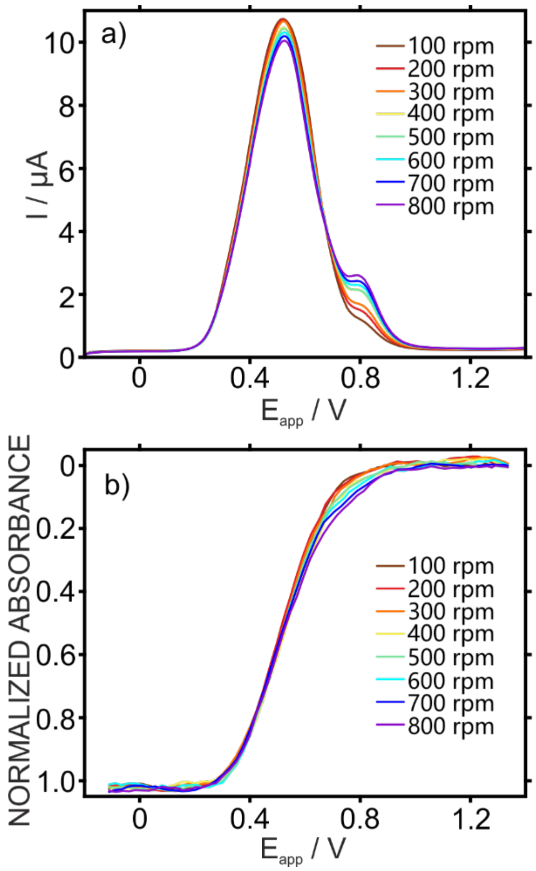

Figure S10. Accumulation/stripping protocol optimization: (a) Stripping voltammograms and (b) the associated dynamic absorbance curves for 10 nM KCl in 10 mM NaCl solution at increasing stirring speeds of 100, 200, 300, 400, 500, 600, 700 and 800 rpm for the accumulation step. Electrochemical protocol: E_{app}=–0.2 V during 700s, linear sweep stripping from –0.2 to 1.4 V, scan rate of 50 mV s^{-1}.

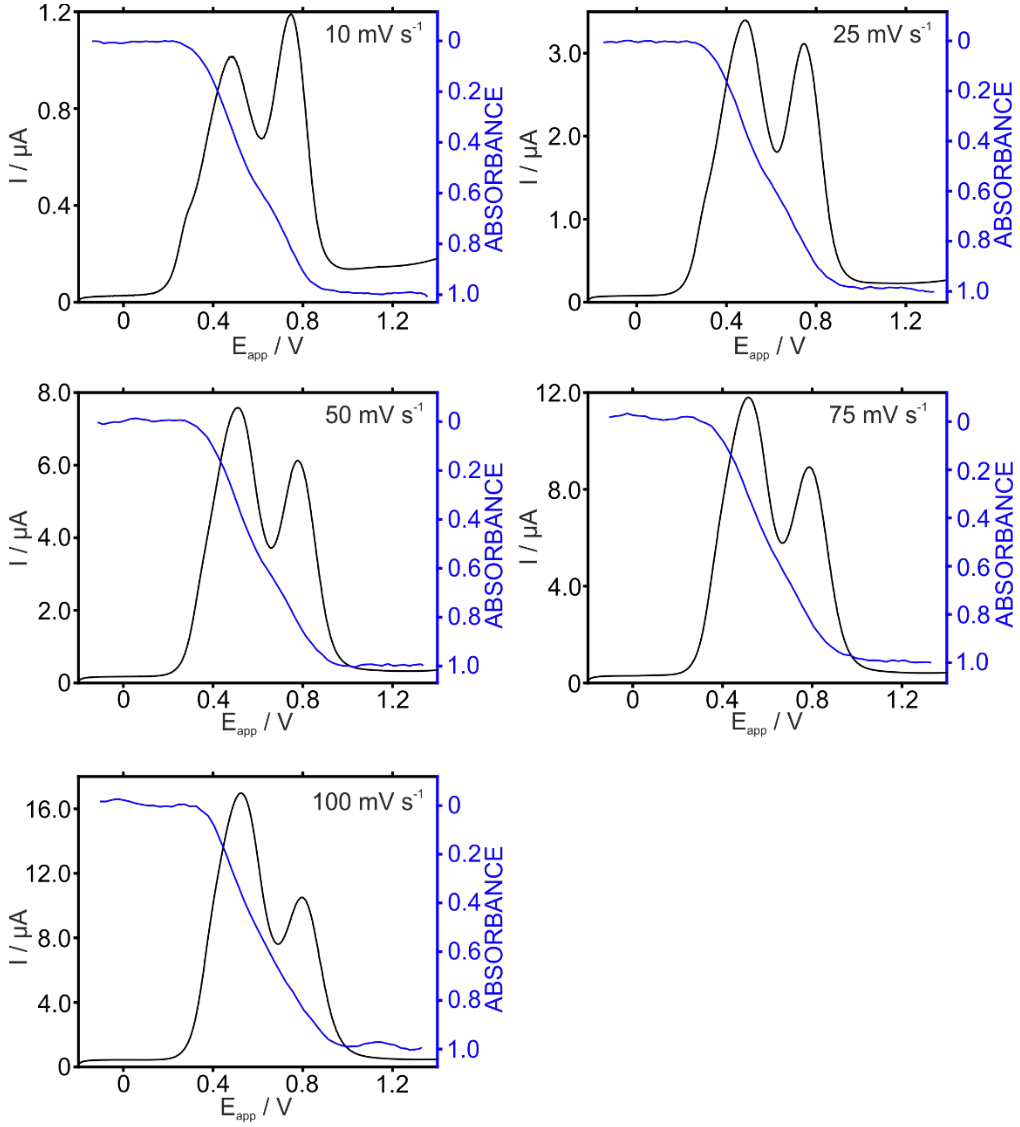

Figure S11. Accumulation/stripping protocol optimization: Stripping voltammograms and the associated dynamic absorbance curves for 450 nM KCl in 10 mM NaCl solution at increasing scan rates of 10, 25, 50, 75, 100 mV s^{-1}. Electrochemical protocol: E_{app}=–0.2 V during 700s, stirring speed of 400 rpm, linear sweep stripping from –0.2 to 1.4 V.

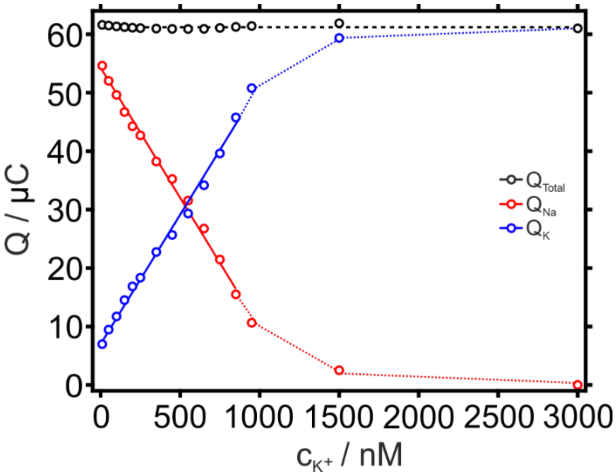

Figure S12. Plot of the peak charge corresponding to the Na^+ and K^+ voltammetric peaks in the experiment in **Figure 4** (main manuscript). The sum of both charges versus the K^+ concentration is also presented. Linear fittings: $Q_{Na^+}(\mu C) = -4.45 \times 10^{-2} c_K(nM) + 54.282, R^2 = 0.9949$ and $Q_{K^+}(\mu C) = 4.43 \times c_K(nM) + 6.992, R^2 = 0.9937$.

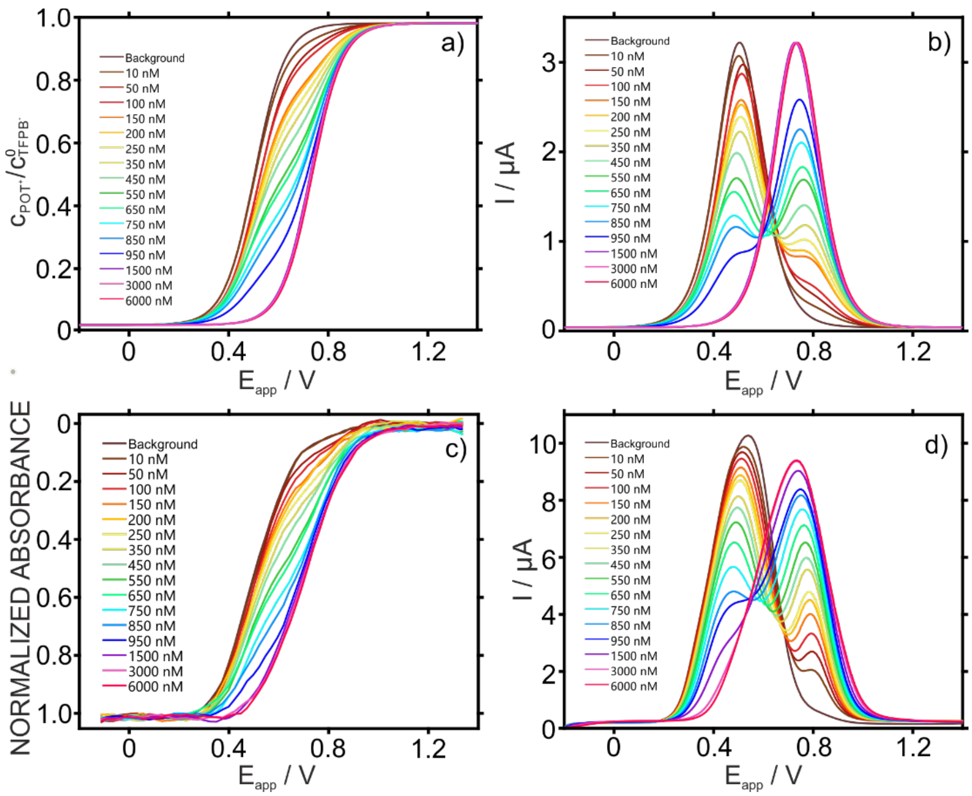

Figure S13. For increasing KCl concentrations at the nanomolar level in 10 mM NaCl background solution: (a) calculated $\frac{c_{POT^+}}{c^0_{TFPB^-}}$ profiles, (b) calculated voltammograms, (c) experimental dynamic absorbance and (d) experimental voltammograms of the POT film. Electrochemical protocol: E_{app}=–0.2 V during 700s, stirring speed of 400 rpm, linear sweep stripping from –0.2 to 1.4 V, scan rate of 50 mV s^{-1}. Notably, the experimental results (c and d) are added for comparative purposes.

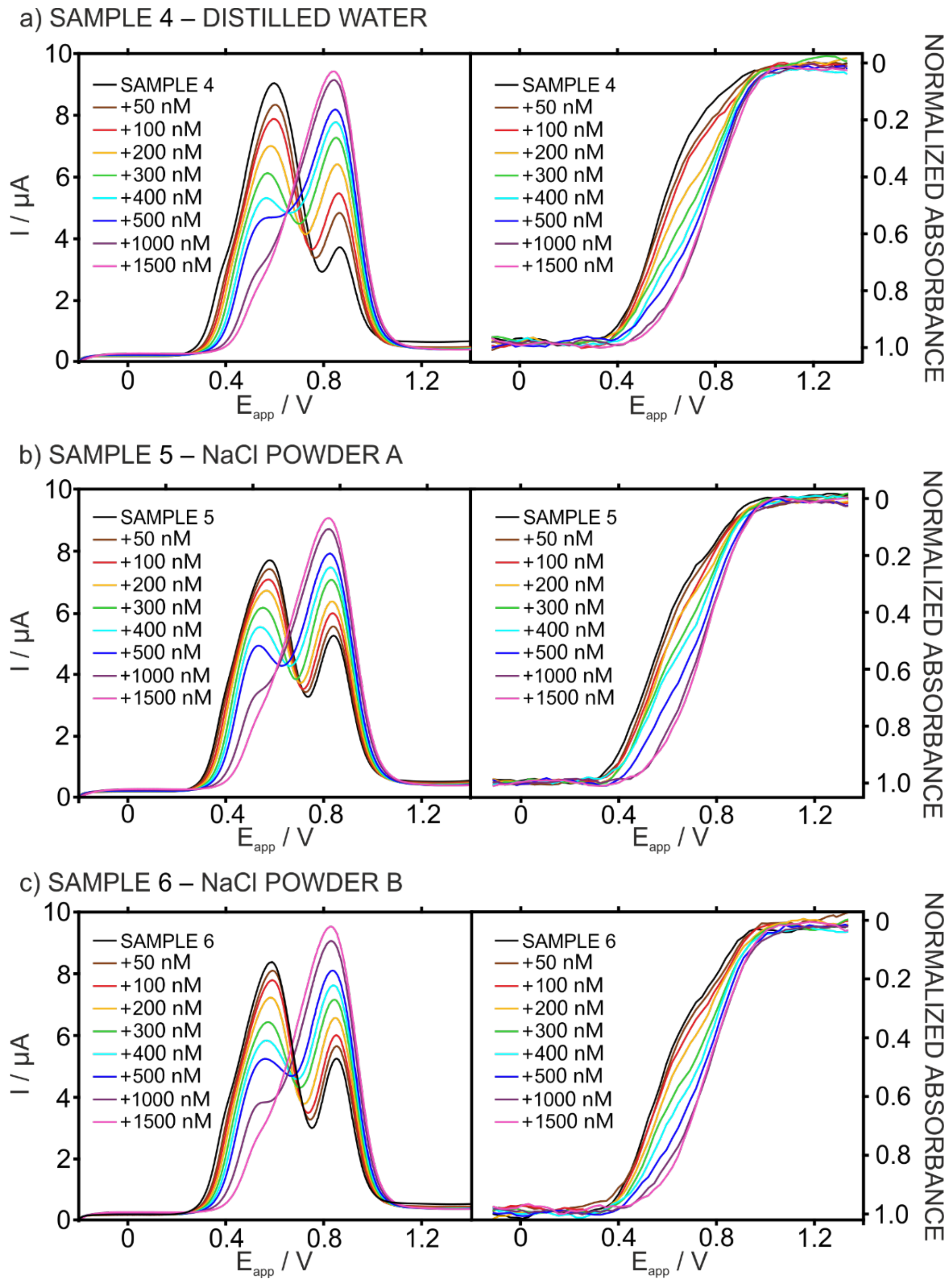

Figure S14. Cyclic voltammograms and dynamic absorbance curves for increasing concentrations of KCl in sample 4 (distilled water), sample 5 (NaCl purchased from Sigma) and sample 6 (NaCl purchased from VWR). Electrochemical protocol: E_{app}=–0.2 V during 700s, stirring speed of 400 rpm, linear sweep stripping from –0.2 to 1.4 V, scan rate of 50 mV s^{-1}.
